# Coverage Optimization Strategy for Wireless Sensor Networks Based on Improved Northern Goshawk Optimization Algorithm

**DOI:** 10.3390/biomimetics11060378

**Published:** 2026-05-31

**Authors:** Shuxin Wang, Yonglong Deng, Nuomei Lan, Li Cao, Zihao Cheng, Mengji Xiong

**Affiliations:** 1 School of Intelligent Manufacturing, Shanghai Zhongqiao Vocational and Technical University, Shanghai 201514, China; 2 Automotive Engineering, Shanghai Food Science and Technology School, Shanghai 201599, China; 3 School of Electronic and Electrical Engineering, Wenzhou University of Technology, Wenzhou 325035, China; 4 College of Control Science and Engineering, Zhejiang University, Hangzhou 310027, China

**Keywords:** wireless sensor networks, coverage optimization, northern goshawk optimization, Tent chaotic map, Gaussian–Lévy mutation

## Abstract

Coverage optimization of wireless sensor networks (WSNs) faces challenges such as uneven node distribution and vulnerability to coverage blind spots. This paper introduces and improves the Northern Goshawk Optimization (NGO) algorithm: the Logistic chaotic map is adopted to initialize the population for enhanced ergodicity, a nonlinear dynamic weight is introduced to balance global exploration and local exploitation, and a Gaussian–Lévy hybrid mutation mechanism is integrated to strengthen the ability to escape from local optima. Experiments on standard test functions show that the improved algorithm (INGO) can stably approach the theoretical optimal values for both unimodal and multimodal functions. The convergence speed and solution accuracy are significantly superior to those of the original NGO, with a smaller standard deviation and stronger robustness. INGO is applied to the coverage optimization of 2D and 3D WSNs, with coverage rate as the fitness function, and the optimal node deployment coordinates are output through iterative optimization. Simulation results show that INGO achieves a best coverage rate of 98.32% in the 2D scenario, which is 7.72 percentage points higher than the 90.6% of NGO. In the 3D scenario, the best coverage rate reaches 72.32%, 6.78 percentage points higher than the 65.54% of NGO. Meanwhile, INGO yields more uniform node deployment and effectively reduces coverage blind spots. Its convergence curve is smooth and oscillation-free in the late iteration stage, and the stability is significantly better than that of NGO. With proper settings of population size and iteration times, INGO can achieve better coverage performance, providing a reliable technical solution for the efficient deployment of wireless sensor networks in complex environments.

## 1. Introduction

With the rapid development of the Internet of Things, 5G communication, artificial intelligence, and other technologies, wireless sensor networks (WSNs) have become key infrastructure connecting the physical world and the digital world [1,2]. By deploying a large number of low-cost and low-power sensor nodes, WSNs collect environmental parameters, transmit data, and perform distributed processing in the target area [3,4]. They are widely used in critical fields such as environmental monitoring, forest fire warning, smart agriculture, the industrial Internet of Things, and urban public safety [5]. In the actual deployment and operation of WSNs, coverage control technology is the core link that determines network performance. Its goal is to maximize the coverage of the monitoring area by optimizing the deployment positions and working states of nodes under the constraints of limited node resources, while reducing sensing blind spots, cutting node redundancy and energy consumption, and extending the network lifetime [6,7].

However, WSN coverage optimization is essentially a multi-constrained, nonlinear, and discrete combinatorial optimization problem, facing many technical challenges [8,9]. First, complex environmental factors (e.g., terrain obstacles, signal attenuation, environmental interference) distort the sensing range of sensors, making traditional coverage strategies based on ideal models difficult to adapt [10]. Second, limited node resources (finite energy, computing capacity, and storage capacity) require coverage strategies to be energy-aware, balancing coverage quality and energy efficiency. Third, dynamic scenarios (e.g., node failure, mobile nodes, changes in monitoring areas) demand adaptive adjustment of coverage strategies to ensure stable and reliable network coverage [11,12]. Traditional coverage control algorithms (e.g., grid deployment, Voronoi diagram, greedy algorithm) often suffer from incomplete coverage, low optimization accuracy, and poor robustness when dealing with the above complex situations, failing to meet practical application requirements [13,14]. As a class of heuristic optimization methods inspired by biological group behaviors in nature, swarm intelligence optimization algorithms have become the mainstream technical approach to solve WSN coverage optimization due to their strong global search ability, independence of gradient information, and good robustness [15,16]. WSN coverage optimization is characterized by discreteness, multi-objectiveness, and strong constraints, which are significantly different from traditional continuous function optimization. By studying the adaptability of the Northern Goshawk Optimization (NGO) algorithm in WSN coverage optimization and designing targeted improvement strategies and constraint handling mechanisms, this paper provides new theoretical references and methodological support for the application of swarm intelligence algorithms in similar engineering problems.

Boasting distributed collaboration, global optimization, and strong robustness, swarm intelligence optimization algorithms have become core and popular technologies in intelligent optimization, widely applied in various complex engineering scenarios [17,18]. In intelligent manufacturing, these algorithms are used for process parameter optimization, production scheduling, and structural design, realizing cost reduction and efficiency improvement under multiple constraints [19,20]. Power and energy systems rely on them to complete grid dispatching, renewable energy allocation, and load optimization, ensuring stable grid operation. In communication, they optimize base station layout, spectrum allocation, and network routing to enhance transmission efficiency and system stability [21]. Meanwhile, they are applied to hyperparameter tuning and feature selection in artificial intelligence, and are rapidly deployed in smart agriculture, satellite planning, and financial portfolio optimization [22,23]. With excellent adaptability to nonlinear, multi-objective, and dynamic optimization problems, swarm intelligence has become a key tool for solving large-scale system optimization, continuously driving the application and development of intelligent optimization technologies [24,25]. In recent years, scholars have proposed various novel swarm intelligence optimization algorithms, such as Particle Swarm Optimization (PSO) and the Grey Wolf Optimizer (GWO), which have been successfully applied to WSN coverage optimization. Proposed in 2021, the Northern Goshawk Optimization (NGO) algorithm is a new swarm intelligence algorithm that simulates the prey recognition and pursuit behaviors of northern goshawks during hunting [26]. It features simple parameter settings, fast convergence, and strong local search capability, and has shown excellent performance in function optimization and engineering design [27,28]. However, its application in WSN coverage optimization is still in the initial stage. The adaptability between the algorithm characteristics and the multi-constraint requirements of WSN coverage optimization needs in-depth research, and the improvement directions and application strategies of the algorithm remain to be further explored.

The major innovations and contributions of this paper are summarized as follows:

(1) Algorithm improvement innovation. To solve the disordered population distribution and premature convergence of original Northern Goshawk Optimization, an improved INGO with multi-strategy fusion is constructed. Tent chaotic initialization, nonlinear dynamic weight, and Gaussian–Lévy mutation are combined to optimize population quality, balance search ability, and avoid local optimum, which effectively enhances convergence accuracy and stability.
(2) Deployment strategy innovation. Combined with the spatial coverage characteristics of wireless sensor networks, the Voronoi diagram mechanism is embedded. The cell centroid is used to dynamically correct node coordinates, reduce coverage overlap and blind areas, and improve node distribution uniformity as well as coverage performance in both two-dimensional and three-dimensional environments.
(3) Experimental system innovation. A multi-level simulation framework is established in this paper. Comparative tests are completed based on benchmark functions and typical WSN scenarios. Statistical significance tests scientifically verify the algorithm’s superiority, providing a feasible reference for the engineering application of swarm intelligence algorithms in sensor network optimization.

## 2. Northern Goshawk Optimization Algorithm

Inspired by the predation behavior of northern goshawks, NGO treats each goshawk as an individual in the population and abstracts predation into two stages [29]. The first stage is exploring and attacking prey, which reflects group behavior by identifying the position of the current optimal solution and guiding each individual to update its position based on the optimal solution, representing global exploration capability. The second stage is prey escape behavior, symbolizing local exploitation capability [30,31].

### 2.1. Algorithm Initialization

The NGO algorithm regards each northern goshawk as an individual (i.e., a feasible solution) and searches for the optimal value in the feasible solution space through movement [32]. Similar to most swarm intelligence algorithms, NGO generates the initial population by random initialization. In the mathematical model, each individual is represented as a D-dimensional vector, and *N* individuals constitute the entire population, which is an *N* × *D* matrix. The mathematical model of the population is given by Equation (1):

(1) $$X={\left[\begin{array}{c} X_{1}\\ \vdots \\ X_{i}\\ \vdots \\ X_{N} \end{array}\right]}_{N\times M}={\left[\begin{array}{ccccc} x_{1,1} & \cdots & x_{1,j} & \cdots & x_{1,M}\\ \vdots & \ddots & \vdots & \ddots & \vdots \\ x_{i,1} & \cdots & x_{i,j} & \cdots & x_{i,M}\\ \vdots & \ddots & \vdots & \ddots & \vdots \\ x_{i,1} & \cdots & x_{i,j} & \cdots & x_{i,M} \end{array}\right]}_{N\times M}$$

where X denotes the population, $X_{i}$ denotes the position of the i-th individual, $x_{i,j}$ denotes the value in the j-th dimension, and M is the maximum dimension of an individual. The objective function is represented by the fitness function.

### 2.2. Prey Recognition and Attack

Prey search and attack form the first stage of each NGO iteration. This stage simulates the optimal solution as prey, with each individual launching an attack on it. In this stage, each individual recognizes the position of the current best solution and updates its own position based on this optimal solution. The main purpose is to enable northern goshawk individuals to search the feasible solution space more extensively for global exploration. Equations (2)–(4) show the mathematical model of the initial stage [33]:

(2) $$P_{i}=X_{Z}$$

(3) $$x_{i,j}^{new1}=\left\{\begin{array}{l} x_{i,j}+r\left(p_{i,j}-I\times x_{i,j}\right),F_{p_{i}}<F_{i}\\ x_{i,j}+r\left(x_{i,j}-p_{i,j}\right),F_{p_{i}}\geq F_{i} \end{array}\right.$$

(4) $$X_{i}=\left\{\begin{array}{l} x_{i}^{new1},F_{i}^{new1}<F_{i}\\ X_{i},F_{i}^{new1}\geq F_{i} \end{array}\right.$$

where i=1,2,…,N, I takes a value of 1 or 2, r=rand(0,1), $P_{i}$ is the candidate solution, $F_{P_{i}}$ is its objective function value, $x_{i}^{new1}$ is the new position in the first stage, $x_{i,j}^{new1}$ is its j-th dimension, and $F_{i}^{new1}$ is the objective function value in the first stage.

### 2.3. Chase and Escape Operation

The second stage is prey chase and escape, which performs local exploitation by simulating the local movement of prey to affect the positions of northern goshawk individuals [34]. The position update formulas in the second stage are shown in Equations (5) and (6):

(5) $$x_{i,j}^{new2}=x_{i,j}+R\left(2r-1\right)x_{i,j}$$

(6) $$X_{i}=\left\{\begin{array}{l} x_{i}^{new2},F_{i}^{new2}<F_{i}\\ X_{i},F_{i}^{new2}\geq F_{i} \end{array}Z=1,2,\cdots I,\cdots N\right.$$

where $x_{i}^{new2}$ is the new position in the second stage, $x_{i,j}^{new2}$ is its value in the j-th dimension, $F_{i}^{new2}$ is the objective function value in the second stage, and R is the hunting radius, expressed as Equation (7) [35]:

(7) $$R=0.02\left(1-\frac{t}{T}\right)$$

where t is the current iteration number and T is the maximum iteration number. R decreases with the increase in iterations, indicating that the hunting radius of the NGO shrinks in the late iteration stage to enhance the local exploitation capability of the algorithm. This mechanism is key to the superior optimization accuracy of NGO compared with many other algorithms [36]. Each iteration of NGO includes these two stages. The current optimal solution acts as a beacon to guide other individuals toward better results.

Northern Goshawk Optimization (NGO) is a novel swarm intelligence optimization algorithm proposed in 2021, which originates from the natural hunting behavior of northern goshawks in nature. Differently from other metaheuristic algorithms with complex evolutionary mechanisms, NGO has a simple structural framework, fewer hyperparameters, and clear optimization logic. The entire hunting process of northern goshawks is abstracted into two core stages: the prey recognition and attack stage, as well as the prey chase and escape stage. This dual-stage iterative mechanism enables the algorithm to alternately complete global exploration and local exploitation during the optimization process. In the initial stage of the algorithm, the population is randomly generated within the search space, and each individual represents a feasible solution corresponding to the sensor node deployment coordinate in WSN. All individuals continuously update their spatial positions by simulating the predation behavior of goshawks, so as to gradually approach the optimal solution in the complex search domain. In the prey recognition and attack phase, the optimal individual in the current population is regarded as the prey. Other individuals learn from the optimal position and carry out wide-range spatial searching, which helps the algorithm excavate potential high-quality solutions in the early iteration and enhances global exploration capability. In the subsequent chase-and-escape phase, the prey actively escapes, and the goshawk adjusts its moving radius to conduct refined local searching around the candidate solution. This stage effectively strengthens the local exploitation ability and improves the optimization accuracy near the optimal solution.

In terms of the parameter iteration mechanism, the hunting radius dynamically decreases with the increase in iteration times, which makes the search range gradually shrink from large-scale global traversal to small-scale local mining. This inherent iteration characteristic enables the original NGO to naturally balance searching intensity without relying on complex manual parameter adjustment. Nevertheless, the traditional NGO still has some inherent defects when solving WSN coverage optimization problems. The random population initialization leads to uneven initial distribution, the fixed weight mechanism lacks adaptive adjustment ability, and the single update rule easily causes premature convergence in complex multi-peak optimization spaces. These shortcomings restrict the further application of NGO in three-dimensional deployment, dense node arrangement, and complex coverage optimization scenarios, which provides a reasonable research motivation and improvement direction for the construction of the improved INGO algorithm in this paper.

## 3. Mathematical Model of Coverage for WSN

This paper constructs two-dimensional and three-dimensional mathematical models for wireless sensor network (WSN) coverage optimization. The classical disc sensing model is adopted as the basic modeling framework, in which all sensor nodes are assumed to have homogeneous sensing and communication radii and are distributed in the form of coordinate points within the continuous monitoring area. Based on the grid discretization principle, the entire monitoring region is divided into equally spaced discrete grid points. The coverage judgment criterion is defined by identifying whether each grid point falls within the sensing range of sensor nodes, and the global network coverage rate is calculated by the ratio of effectively covered grid points to the total number of grid points. The model introduces Voronoi partition mechanism to divide the monitoring area into multiple adjacent sub-regions corresponding to each sensor node. The geometric centroid of each Voronoi cell is calculated to guide the adaptive migration of sensor nodes toward sparse and blind areas, which effectively reduces coverage overlap, eliminates monitoring blind areas, and improves the uniformity of node deployment. In terms of model constraints, boundary limitation, fixed sensing radius and node quantity constraints are formulated to restrict all nodes within the monitoring scope and avoid invalid cross-boundary deployment [37].

The optimization objectives of the established model are to maximize regional coverage, minimize coverage overlap and blind areas, and maintain balanced spatial distribution of nodes, which conforms to the practical engineering requirements of WSN deployment. Although the model adopts the ideal disc sensing assumption without introducing terrain occlusion, sensing random error and node failure factors, it follows the mainstream modeling paradigm of the swarm intelligence algorithm for WSN optimization, and can provide a standard simulation benchmark and optimal deployment reference for practical sensor arrangement in complex environments. To meet the requirements of WSN coverage optimization, domain knowledge is introduced to guide the rational distribution of nodes and improve coverage efficiency. The specific implementation strategies and core formulas are as follows. A Voronoi diagram guides node repositioning. The centroid of the Voronoi region of each node is calculated periodically to reduce coverage overlap. The centroid formula is [38]:

(8) $$C_{j}=\frac{1}{M}\sum_{k\in V_{j}}G_{k}$$

Nodes move a small step toward the centroid. The movement formula is:

(9) $$N_{j}^{\mathrm{new}}=N_{j}+\frac{C_{j}-N_{j}}{∥C_{j}-N_{j}∥}\cdot 0.5$$

The grid discretization method is used to judge the coverage state. The coverage state formula is [39]:

(10) $$\mathrm{cover}(G_{k})=\{\begin{array}{cc} 1, & \exists j,∥G_{k}-N_{j}∥\leq R_{s}\\ 0, & \mathrm{otherwise} \end{array}$$

A small-scale Gaussian perturbation search is performed on the global optimal individual. The formula is:

(11) $$X_{\mathrm{local}}=\mathrm{BestPos}+0.005\cdot \left(ub-lb\right)\cdot N\left(0,1\right)\cdot \left(1-\frac{t}{T}\right)$$

When there is no update, 5% of the worst individuals are restarted. The restart formula is [40]:

(12) $$X_{\mathrm{restart}}=lb+\mathrm{rand}\cdot \left(ub-lb\right)$$

All individuals must satisfy boundary constraints to ensure solution validity:

(13) $$X=max\left(min\left(X,ub\right),lb\right)$$

## 4. Improved Northern Goshawk Optimization Algorithm

Aiming at the problems of uneven node coverage, coverage blind spots, and premature convergence of traditional optimization algorithms in wireless sensor networks (WSNs), an improved multi-strategy fusion algorithm (INGO) is proposed based on the traditional NGO. The improvement strategies run through the whole process of initialization, adaptive parameter adjustment, position update, WSN scenario guidance, and population stagnation intervention. The specific design is as follows.

### 4.1. Hybrid Initialization Strategy

Random initialization in traditional algorithms easily leads to uneven population distribution and slow early convergence [41]. To improve the quality of initial solutions and enhance ergodicity, a hybrid initialization of chaotic map, opposition-based learning, and uniform grid is adopted, and the initial population is constructed by optimal selection.

(1) Logistic chaotic map generates uniformly ergodic chaotic sequences to enhance the diversity of the initial population [42]:

(14) $$x_{i+1}=4\cdot x_{i}\cdot \left(1-x_{i}\right)$$

The chaotic sequence to the solution space is mapped:

(15) $$X_{\mathrm{chaos}}=lb+x\cdot \left(ub-lb\right)$$

(2) Opposition-based learning generates opposite solutions for chaotic solutions to further expand the search space:

(16) $$X_{\mathrm{oppo}}=lb+ub-X_{\mathrm{chaos}}$$

(3) Uniform grid initialization generates spatially more uniform initial individuals combined with the characteristics of WSN node distribution. Finally, the three types of initial solutions are merged to evaluate fitness, and the optimal *N* individuals are selected as the initial population to improve the starting quality of the algorithm [43].

### 4.2. Nonlinear Dynamic Parameter Adjustment Strategy

To reasonably balance the global exploration and local exploitation capabilities of the Northern Goshawk Optimization algorithm during iterations, a Sigmoid-type nonlinear dynamic weight function is adopted for adaptive parameter adjustment in this paper. Differently from the traditional linearly decreasing weight, the Sigmoid nonlinear weight possesses smooth, nonlinear and controllable variation characteristics, which is more consistent with the iterative search law of intelligent optimization algorithms. In the early iteration stage, the weight maintains a relatively high value. This design retains a larger individual displacement step, expands the population search range, enables the algorithm to traverse the global space extensively, and avoids premature convergence and local optimum trapping. In the middle iteration stage, the weight decreases smoothly following the S-shaped curve, realizing a steady transition from global exploration to local exploitation and preventing search oscillation caused by sudden parameter changes. In the late iteration stage, the weight converges to a low value and reduces the amplitude of individual position updates, so that the algorithm can conduct fine exploitation near the optimal solution and improve local search accuracy.

From the perspective of WSN engineering optimization, sensor node deployment belongs to a complex nonlinear spatial optimization problem. Fixed weight or linear weight is difficult to adapt to the search rhythm of complex monitoring spaces. The adopted nonlinear weight function can adaptively adjust the attenuation rate according to the iterative evolution state without frequent manual parameter tuning, which can dynamically match the node distribution optimization requirements of two-dimensional and three-dimensional sensing regions. This weight function has no complex hyperparameters, a low computational cost, and a smooth convergence curve. Compared with step weight and linear weight, it is more compatible with the two-stage hunting update mechanism of the Northern Goshawk Optimization algorithm. It reduces the probability of premature convergence from the mechanism level and provides a stable and excellent iterative search foundation for subsequent improvement strategies such as Gaussian–Lévy mutation and Voronoi centroid optimization [44]. The Sigmoid nonlinear inertia weight realizes smooth decay through an S-curve. This design enables the algorithm to have a high inertia weight in the early stage, focusing on global exploration to expand the search space and avoid falling into local optima. In the later stage, the inertia weight gradually decreases, focusing on local exploitation to refine the solution quality, effectively avoiding excessive early oscillation and insufficient later exploitation. Its expression is:

(17) $$w=w_{min}+\frac{w_{max}-w_{min}}{1+\exp\left(10\left(\frac{t}{T}-0.5\right)\right)}$$

where $w_{max}=0.95$ and $w_{min}=0.25$ are the maximum and minimum values of the inertia weight, respectively. t is the current iteration number and T is the maximum iteration number. The decay rate of the inertia weight is dynamically regulated by the iteration ratio [45]. Meanwhile, the nonlinear decreasing learning factor adopts a square-root nonlinear decay strategy. Its core purpose is to gradually reduce the learning intensity with the iteration process. The learning factor is large in the early stage to accelerate convergence, and small in the later stage to avoid oscillation near the optimal value. Its expression is:

(18) $$c_{1}=c_{1max}\cdot {\left(1-\frac{t}{T}\right)}^{0.5}$$

where $c_{1max}=0.8$ is the initial maximum value of the learning factor, ensuring strong learning ability in the early stage and gradual decay in the later stage for fine optimization.

### 4.3. Two-Stage Position Update and Hybrid Mutation Strategy

To balance global exploration and local exploitation and avoid local optima, INGO adopts two-stage update of prey recognition + chase escape, and introduces Gaussian–Lévy hybrid mutation to improve escape ability.

The prey recognition stage updates differentially according to fitness:

(19) $$X_{\mathrm{new}}=X_{i}+w\cdot r\cdot \left(P_{i}-I\cdot X_{i}\right)+c_{1}\cdot \mathrm{rand}\cdot \left(\mathrm{BestPos}-X_{i}\right)$$

(20) $$X_{\mathrm{new}}=X_{i}+w\cdot r\cdot \left(X_{i}-P_{i}\right)+c_{1}\cdot \mathrm{rand}\cdot \left(\mathrm{BestPos}-X_{i}\right)$$

where I∈1, 2 is a random integer, r is a random vector in [0, 1], w and $c_{1}$ are adaptive weight and learning factor, respectively, and BestPos is the global optimal position. The chase and escape stage is the core of algorithm exploitation, focusing on optimizing potential high-quality solutions from the prey recognition stage. An adaptive attraction factor that dynamically decays with iterations is introduced:

(21) $$R_{\mathrm{att}}=0.02\cdot \left(1-\frac{t}{T}\right)$$

The basic position update formula is

(22) $$X_{\mathrm{new}}=X_{i}+R_{\mathrm{att}}\cdot \left(2r-1\right)\cdot X_{i}$$

Gaussian–Lévy hybrid mutation is triggered with low probability. The Gaussian mutation formula is

(23) $$X_{\mathrm{gauss}}=X_{\mathrm{new}}+\sigma \cdot N\left(0,1\right)$$

where the mutation step size is

(24) $$\sigma =0.002\cdot \left(ub-lb\right)\cdot \left(1-\frac{t}{T}\right)$$

The Lévy flight mutation formula is

(25) $$X_{\mathrm{levy}}=X_{\mathrm{new}}+0.005\cdot L\cdot \left(\mathrm{BestPos}-X_{\mathrm{new}}\right)$$

where the Lévy step size satisfies:

(26) $$L=\frac{u}{|v{|}^{\frac{1}{\beta }}},\beta =1.5$$

### 4.4. Algorithm Complexity Analysis

The time complexity of the Improved Northern Goshawk Optimization (INGO) consists of four parts: population initialization, fitness calculation, position update iteration, and boundary constraint. Let the population size be N, the optimization dimension (number of sensor network nodes × dimension of parameters to be optimized) be D, and the maximum iteration number be T. Population initialization: chaotic map initialization traverses N individuals and D-dimensional parameters, with time complexity O(N×D). Fitness calculation: the coverage fitness of N individuals is calculated in each iteration, the single individual complexity is O(D), and the single iteration complexity is O(N×D). Position update iteration: the algorithm includes the exploration stage (foraging), exploitation stage (attack), adaptive weight update, and Lévy flight perturbation. Each iteration performs a D-dimensional position operation on N individuals, with single iteration complexity O(N×D); the total complexity of T iterations is O(T×N×D). Boundary constraint: executed synchronously with position update, and no additional complexity overhead.

In summary, the total time complexity of INGO is O(T×N×D), which is consistent with that of the original NGO. The improved strategies (chaotic initialization, adaptive weight, Lévy flight) do not increase the time complexity of the algorithm, ensuring the operating efficiency.

From the perspective of the trade-off between computational overhead and optimization performance, the integration of multiple improved modules inevitably increases limited computational consumption. Compared with the basic NGO, the adaptive adjustment mechanism, mutation perturbation strategy, and spatial optimization operator adopted by INGO increase the logical judgment times and search steps during iteration, resulting in minor time loss. Nevertheless, the increased computational cost is within a reasonable and controllable range without exponential complexity growth. Meanwhile, the multi-strategy collaborative improvement significantly optimizes population spatial distribution, balances global exploration and local exploitation, and reduces the risk of premature convergence. The proposed algorithm obtains prominent performance improvements in coverage rate, deployment uniformity, and convergence stability. Comprehensive analysis demonstrates that INGO achieves remarkable performance gain at the cost of negligible computational increment. The optimization improvement is far greater than the computational overhead, presenting an excellent trade-off relationship. The improved algorithm maintains high engineering practicability and is more suitable for node deployment optimization of wireless sensor networks.

### 4.5. Implementation Steps of INGO

The monitoring area of the wireless sensor network is set as a two-dimensional plane, the number of sensor nodes to be deployed is n, and the optimization goal is to maximize the network coverage C while balancing node energy consumption. The Improved Northern Goshawk Optimization (INGO) is used to optimize the node position coordinates. The implementation steps are as follows:

Step 1: Initialize parameters. Set population size N, maximum iterations $T_{max}$, search space bounds ub/lb, adaptive weight parameters $\omega_{max}/\omega_{min}$, Lévy flight parameter β, and learning factors $c_{1},c_{2}$, and define the coverage fitness function.

Step 2: Chaotic population initialization. Use a Sinusoidal chaotic map to generate the initial goshawk population, where each individual corresponds to a set of sensor node position coordinates to ensure uniform distribution of initial solutions.

Step 3: Fitness calculation. Decode each goshawk individual into a node deployment scheme, calculate network coverage and energy balance, and substitute into the fitness function to obtain individual fitness values.

Step 4: Optimal position update. Calculate and record the individual optimal position $P_{best}$ and global optimal position $G_{best}$ of the current iteration.

Step 5: Adaptive weight update. Dynamically calculate inertia weight according to iteration times to balance global exploration and local exploitation.

Step 6: Position update iteration. Select exploration stage (foraging behavior) or exploitation stage (attack behavior) to update positions by probability; introduce Lévy flight in the exploration stage to enhance global search, and adopt adaptive weight and optimal guidance strategy in the exploitation stage.

Step 7: Boundary constraint processing. Restrict out-of-bounds node position coordinates to the monitoring area.

Step 8: Greedy selection update. Compare the fitness of new and original positions, retain better solutions, and update individual positions and optimal information.

Step 9: Iteration termination judgment. If the maximum iterations are reached, output the global optimal node deployment scheme and coverage; otherwise, return to Step 4 to continue iteration.

The pseudocode of the INGO algorithm is shown in Algorithm 1.

**Algorithm 1**: The INGO algorithmInput: *Population size N, maximum iterations Tmax, upper and lower bounds ub, lb of search space, sensing radius Rs*Output: *Global optimal node coordinate BestPos, optimal coverage rate CR_best_*1. Initialize algorithm parameters: inertia weight bounds $w_{\max}$, $w_{\min}$, learning factor *c*_1_, Lévy flight parameter β.
2. Initialize population $X_{i}(i=1,2,\ldots ,N)$ by hybrid strategy of Tent chaotic mapping and opposition-based learning. 3. Calculate WSN coverage fitness value of each individual according to Equation (5). 4. Initialize personal optimum *P_best_*, global optimum *G_best_* and optimal position *BestPos*. 5. for 1 to *Tmax* do 6. Update nonlinear dynamic inertia weight w and learning factor *c_1_* according to Equations (11) and (12). 7. for *I* = to *N* do 8. Prey recognition stage: update individual initial position according to Equations (13) and (14). 9. Chase and escape stage: update position via hunting radius based on Equations (15) and (16). 10. Trigger Gaussian–Lévy hybrid mutation with small probability, and implement mutation according to Equations (17)–(20). 11. Deal with boundary constraints and correct out-of-bound positions according to Equation (26).12. Return $x_{gbest}$, $f\left(x_{gbest}\right)$
13. Greedy selection: update individual position and *P_best_* if the new fitness is better.
14. end for
15. Fine-tune the position of global optimum by Voronoi centroid strategy in Equations (21) and (22).
16. Iteration stagnation judgment: restart 5% worst individuals if no optimal update within 35 consecutive generations (Equation (25)).
17. Update global optimum *G_best_* and optimal coverage rate $CR_{\mathrm{best}}$.
18. end for 19. return *BestPos* and $CR_{\mathrm{best}}$

## 5. Experimental Results and Analysis of Algorithm Testing

### 5.1. Experimental Environment Setup

To fully verify the effectiveness of the proposed INGO in solving optimization problems, multiple groups of comparative experiments are carried out on the MATLAB platform. All algorithms run on a 64-bit Windows 11 operating system with 16 GB RAM, using MATLAB R2023b to ensure a unified experimental environment and reliable results. A variety of mainstream intelligent optimization algorithms are selected as controls, including Particle Swarm Optimization (PSO) [46], the Grey Wolf Optimizer (GWO) [47], the Butterfly Optimization Algorithm (BOA) [48], the Gorilla Troops Optimizer (GTO) [49], the Dung Beetle Optimizer (DBO) [50], Spider Wasp Optimization (SWO) [51], and the original Northern Goshawk Optimization (NGO), for comprehensive comparison with INGO.

The performance of the algorithm is verified through standard test functions and WSN coverage optimization scenarios, and quantitative analysis is carried out from the dimensions of convergence speed, solution accuracy, and stability to fully prove the advantages of INGO in global search, local exploitation, and complex problem optimization, ensuring persuasive and scientific experimental results.

### 5.2. Functional Testing

In the experiment, CEC2017 is selected as the benchmark test suite to comprehensively test the global optimization performance and convergence characteristics of the proposed INGO. The CEC2017 benchmark test suite contains 30 standard test functions, all designed for minimization problems, which can be divided into four categories according to function characteristics: F1–F3: Unimodal functions, used to verify the local exploitation ability and convergence accuracy of the algorithm. F4–F10: Simple multimodal functions, used to evaluate the global search ability and local optimum avoidance ability of the algorithm. F11–F20: Hybrid functions, integrating unimodal and multimodal characteristics, used to test the balance ability of the algorithm in complex search spaces. F21–F30: Composite functions, with high-dimensional, multi-extremum, and strong nonlinear characteristics, used to verify the robustness of the algorithm in extremely complex optimization scenarios. The unified search range of all functions is $[-100,100{]}^{D}$, which can adapt to optimization test requirements of different dimensions.

This test suite covers various optimization scenarios from simple to complex and from low-dimensional to high-dimensional. It can not only comprehensively evaluate the global optimization ability, convergence speed, and solution accuracy of INGO, but also fully measure the adaptability and stability of the algorithm in different types of optimization problems, fully verifying the performance advantages of INGO in solving complex numerical optimization problems, and providing reliable algorithm performance support for practical engineering applications such as subsequent WSN node deployment optimization. Table 1 provides detailed classification and characteristic information of the 30 valid functions in the CEC2017 benchmark test set.

**Figure 1:**
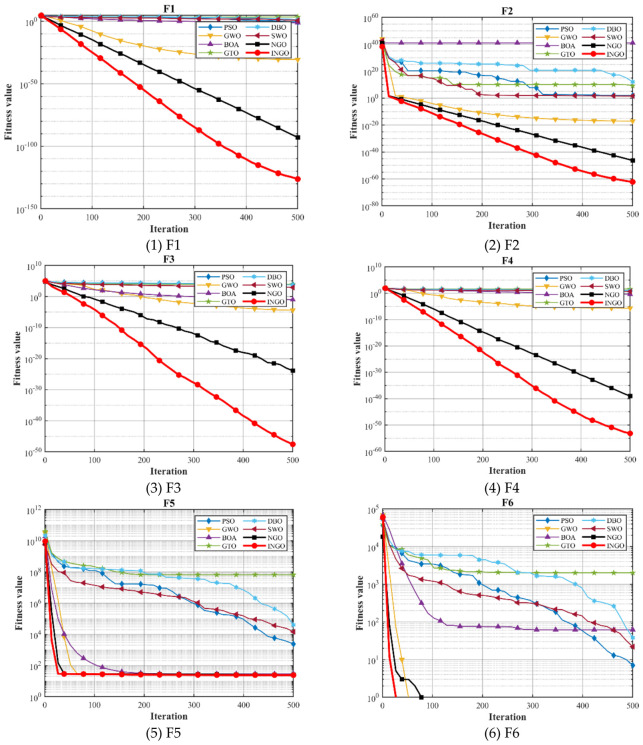

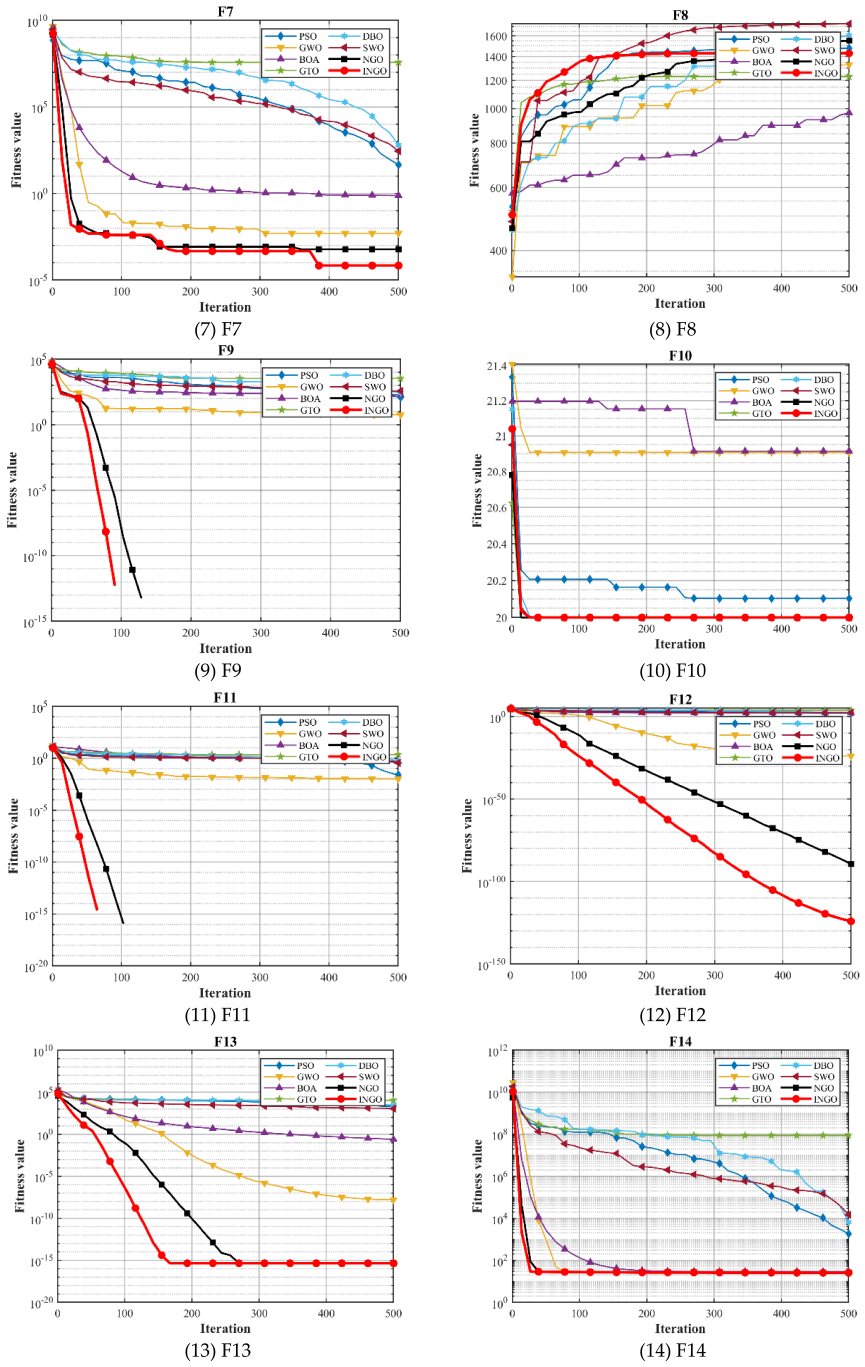

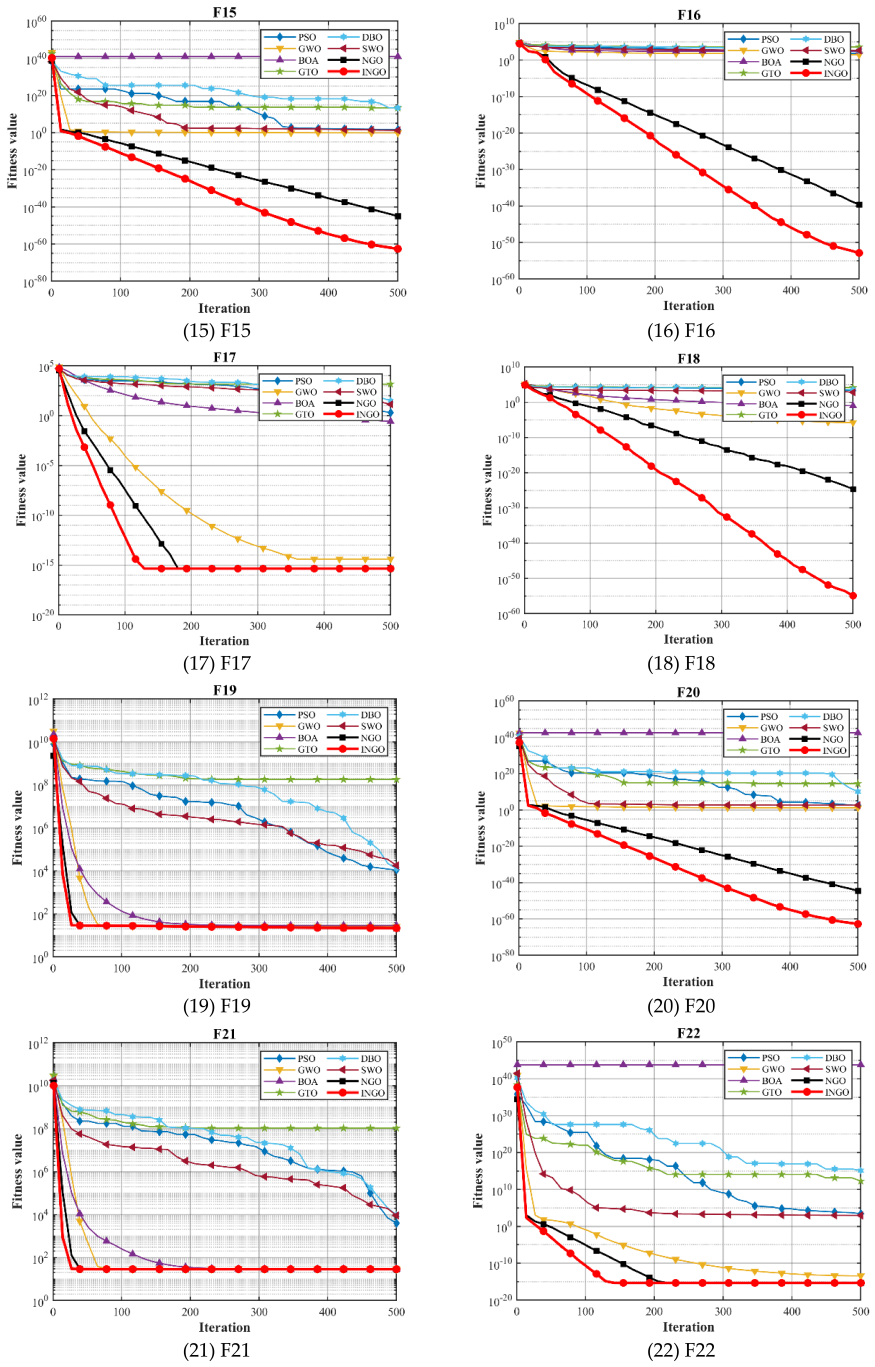

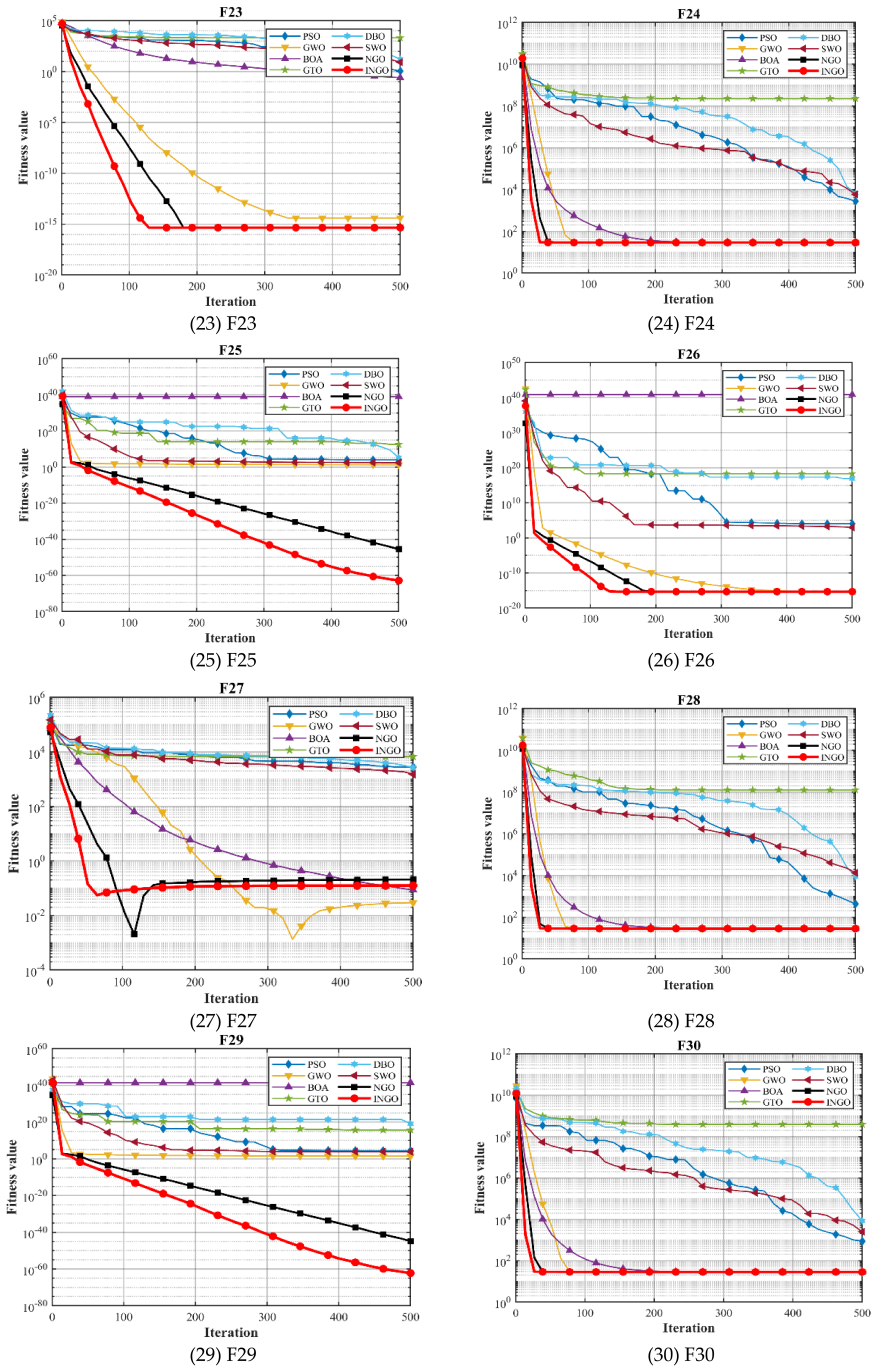
Iterative curves of different improved algorithms (Dim = 30).

To verify the optimization performance and engineering practicability of the proposed INGO, comparative experiments are conducted with a variety of mainstream intelligent optimization algorithms in recent years. The CEC2017 benchmark test suite is used in the experiment, and the test dimension is uniformly set to D=30. Table 1 shows the statistical results of 30 independent runs of each algorithm, giving the optimal fitness value (min), average fitness value (avg), and standard deviation (std) of fitness values, respectively. The bold data represent the optimal performance in this comparison. Figure 1 shows the average convergence curve of each algorithm in the iterative optimization process, which can intuitively reflect the convergence speed and global search ability of the algorithm. Figure 1 shows the average convergence curves of each algorithm during the iterative optimization process, which can intuitively reflect the convergence speed and global search ability of the algorithm. Results of different swarm intelligence algorithms (Dim = 30) is shown in Table 2.

In the evaluation of CEC2017 test functions, INGO performs prominently, obtaining 15 optimal solutions in 30 test functions, fully demonstrating the effectiveness of its optimization strategy. Especially on functions such as F1, F2, F3, and F4, INGO is significantly better than other mainstream algorithms such as PSO, GWO, and BOA, achieving lower best and average values. Although INGO performs slightly weaker in some test functions such as F5, F14, F21, F22, and F23, its results are still close to the optimal values, reflecting its good practicability and stability. INGO integrates a variety of strategies such as chaotic search, Voronoi diagram guidance, and blind spot filling, making it highly adaptable and robust in diverse test environments. In addition, stability analysis shows that INGO has small fluctuations in most test functions, further verifying its reliability in multiple experiments. In summary, INGO shows excellent optimization performance in the CEC2017 test functions, confirming its potential as an effective and practical optimization algorithm worthy of promotion and use in practical applications.

As shown in Figure 1, taking function F4 as an example, both INGO and NGO show a rapid downward trend in the initial stage, but the elite guidance strategy introduced by INGO enables it to approach the global optimal region more accurately in the early iterations. At about 200 iterations, some comparison algorithms such as GWO and DBO slow down significantly after falling into local optima, while INGO successfully jumps out of the local trap and continues to approach better solutions by virtue of the random long jump generated by the Lévy flight mechanism. It can be seen from the convergence curve that INGO has stabilized at the minimum order of magnitude after 200 iterations, while NGO also achieves high accuracy but with a slightly slower speed, and other algorithms stagnate between $10^{-1}$ and $10^{-7}$. This shows that the improved strategy of INGO significantly enhances the ability of the algorithm to get rid of local optima, enabling the improved algorithm to jump out of local optima more quickly and maintain efficient global search in subsequent iterations, and finally obtain the global optimal value.

**Figure 2:**
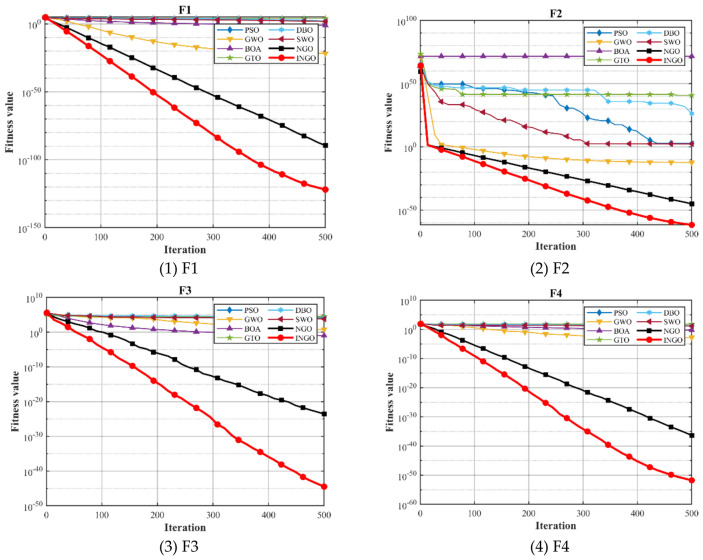

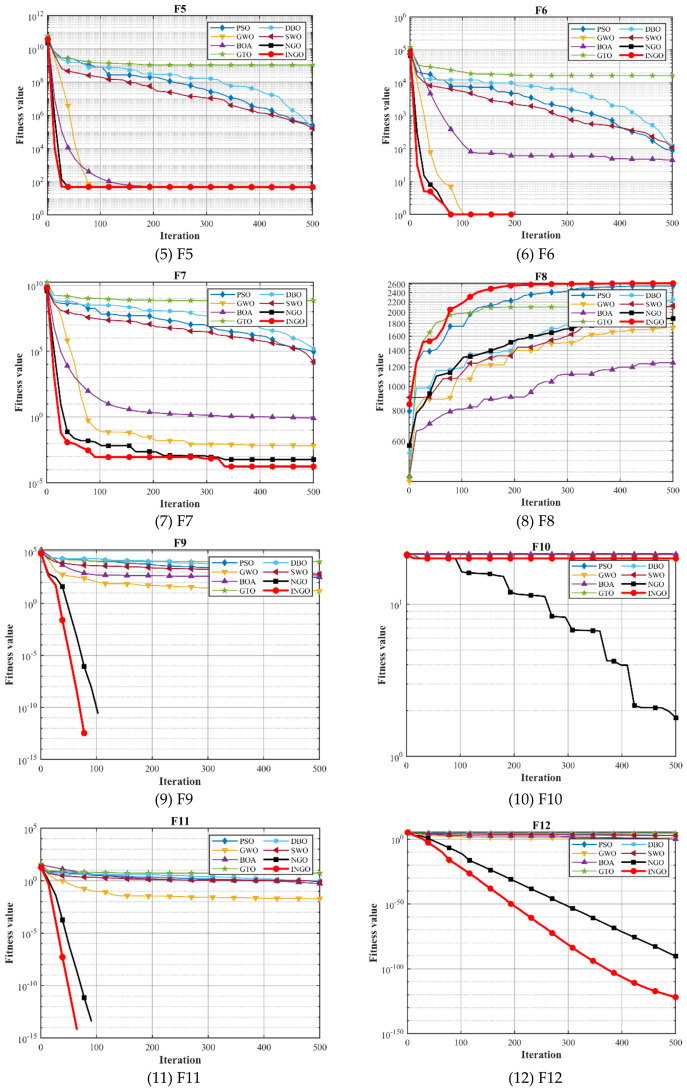

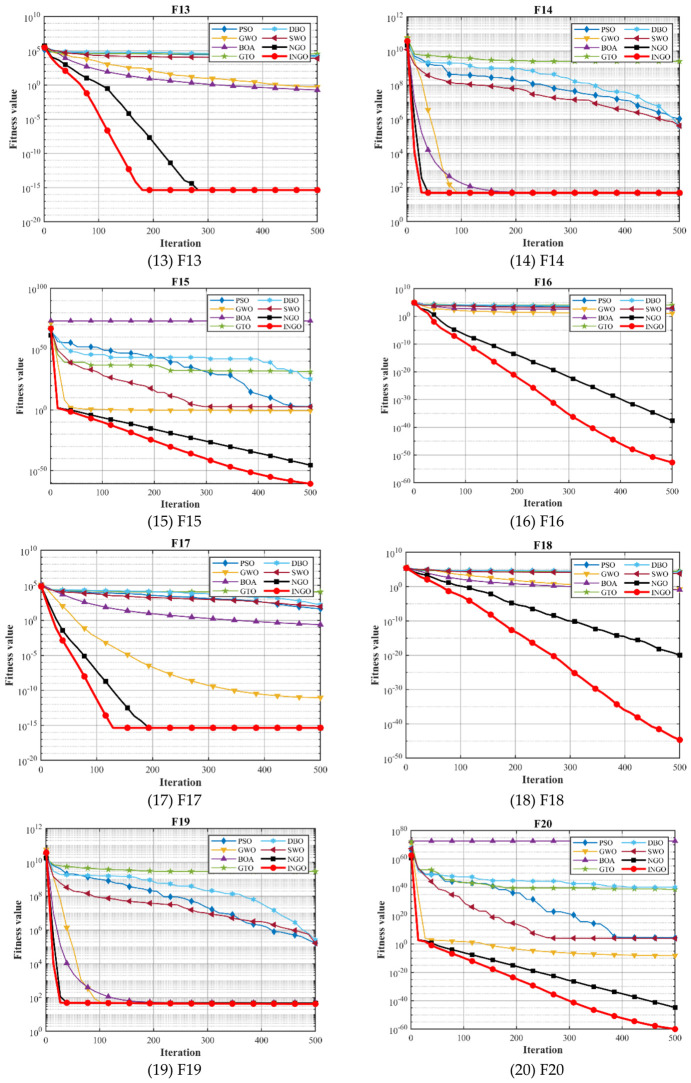

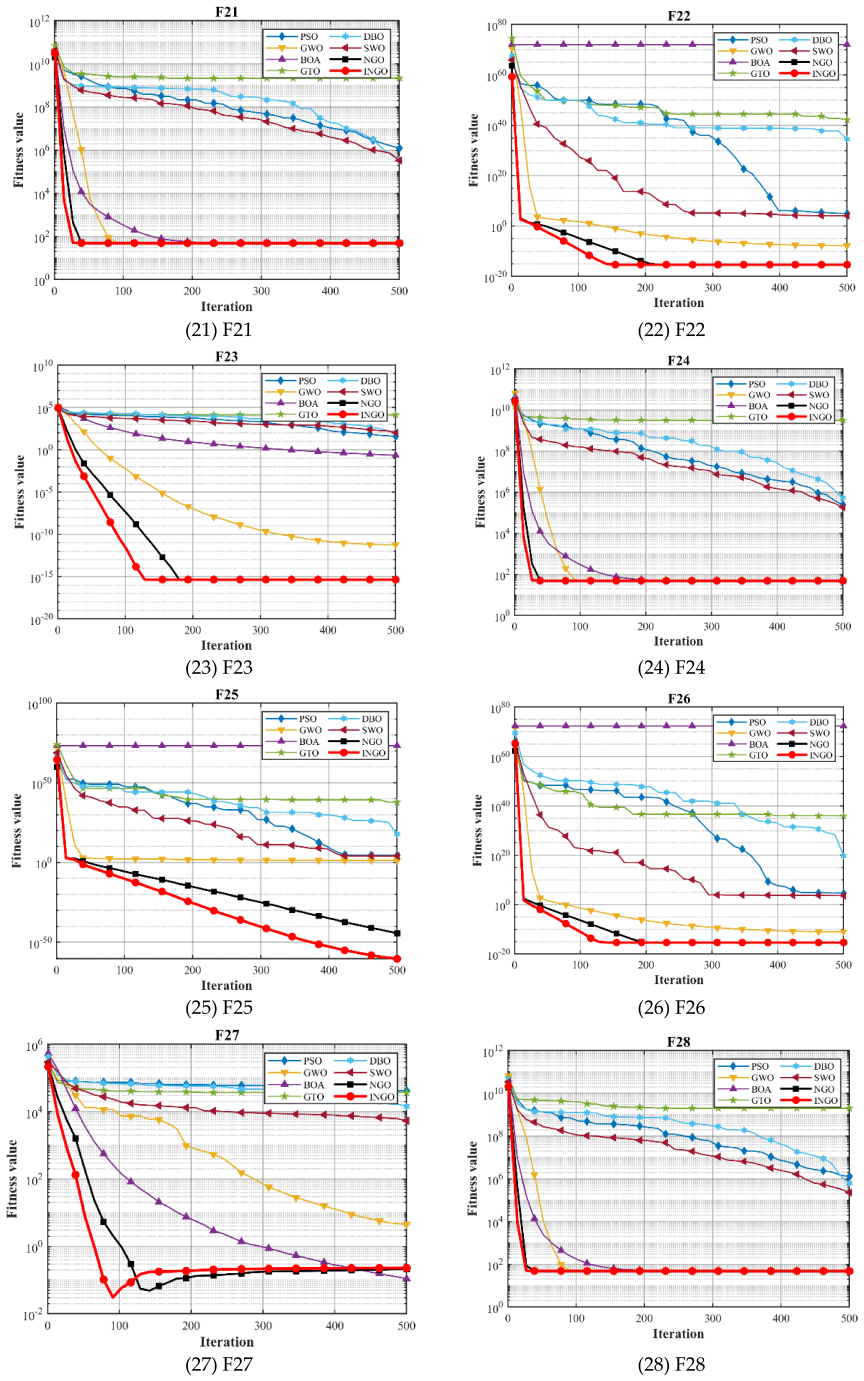

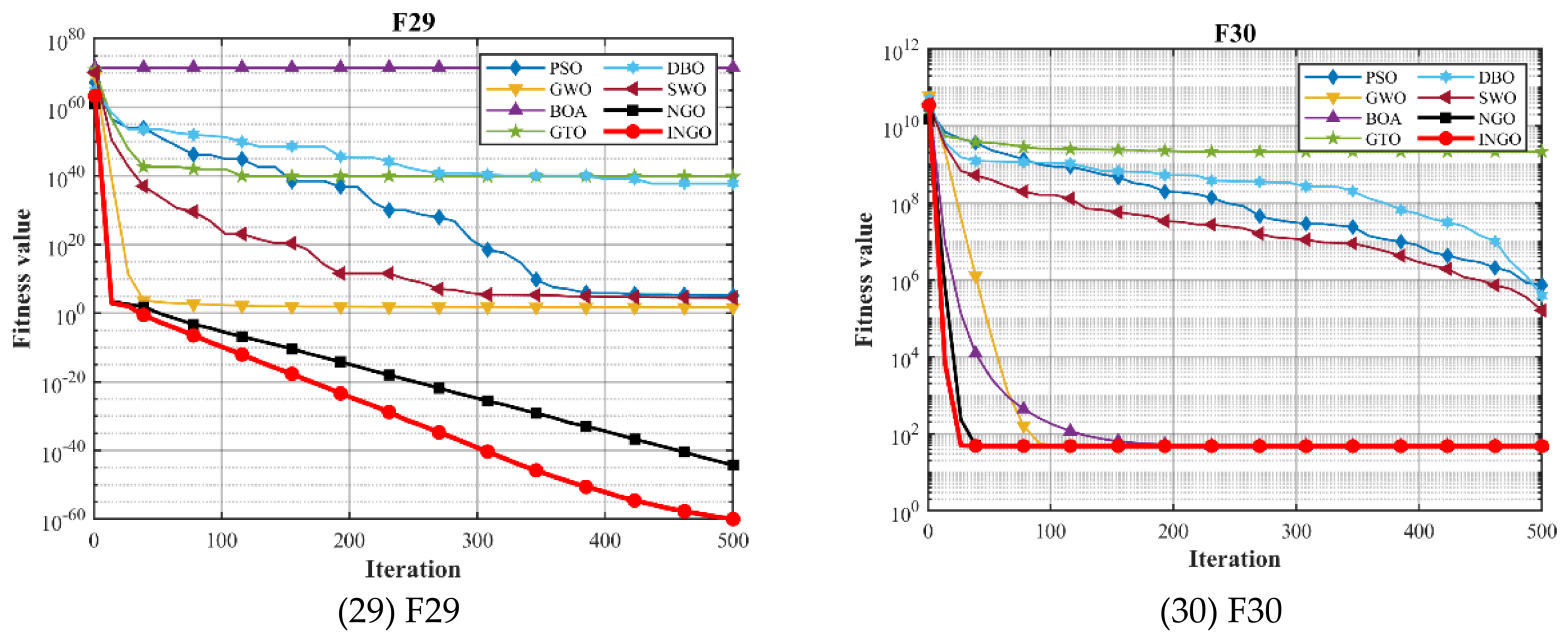
Iteration curve diagrams of different improved algorithms (Dim = 50).

To further verify the feasibility and effectiveness of the proposed improved algorithm in high-dimensional problems, the eight algorithms are further solved in the CEC2017 test set with test dimension Dim=50. Table 3 records the optimal fitness value (min), average fitness value (avg), and standard deviation (std) of fitness values, with bold data indicating the best results. Figure 2 shows the average convergence curve during iteration.

Wilcoxon rank-sum test and Friedman test (Dim = 50). To quantitatively and scientifically evaluate the optimization performance of the proposed algorithm and comparison algorithms, two classical and reliable non-parametric statistical tests, including the Wilcoxon rank-sum test and Friedman test, are utilized in this study. As a mainstream pairwise statistical analysis method, the Wilcoxon rank-sum test is capable of analyzing the significant differences between two independent optimization algorithms without strict data distribution assumptions. In general, the *p*-value serves as the critical evaluation criterion; specifically, a *p*-value lower than the statistical significance threshold of 0.05 demonstrates that evident performance discrepancies exist between the two compared algorithms. Differently, the Friedman test is applied to conduct comprehensive multi-algorithm performance evaluation. This method calculates the average ranking of each competitor algorithm under diverse benchmark functions, thereby providing an intuitive and quantitative comparison of algorithm optimization capability. Notably, a lower average ranking represents superior comprehensive optimization performance and stronger stability. The detailed statistical difference results and average ranking values of all algorithms on the CEC2017 benchmark test functions (Dim = 50) are summarized in Table 4.

Table 4 summarizes the statistical outcomes of the Wilcoxon rank-sum test and Friedman test derived from the experimental data in Algorithm 1. In this table, the discrimination result marked with “Y” or “N” represents whether statistically significant performance differences exist between the proposed INGO and other comparative algorithms. According to the statistical verification results, obvious significant discrepancies can be observed between INGO and all nine baseline algorithms, which demonstrates that the optimization superiority of INGO is statistically credible. Moreover, the average ranking obtained from the Friedman test further quantitatively evaluates the comprehensive performance of each algorithm. The results reveal that INGO achieves the optimal ranking among all comparison methods. Generally, higher average ranking values imply weaker optimization capability and limited improvement effectiveness. Combined with the previous convergence, coverage, and robustness experimental results, it can be conclusively verified that the proposed INGO possesses remarkable competitiveness and superior performance in solving complex optimization problems.

From the results in Table 2, INGO shows excellent performance on almost all test functions, significantly better than comparison algorithms such as PSO, GWO, BOA, GTO, DBO, and SWO, and further improved compared with the original NGO. Specifically, INGO can reach the optimal value of the order of $10^{-64}$ to $10^{-127}$ on functions such as F1, F2, F3, F4, F11, F12, F13, F17, F18, F22, F23, and F26, far better than the local optima or large values common in other algorithms, reflecting a high balance between global search and local exploitation. On discrete or multimodal functions such as F6, F9, and F11, INGO can stably obtain the theoretical optimal value 0 with a very small standard deviation, proving its complete optimization ability and robustness. On functions such as F7, F10, F21, and F24, the standard deviation of INGO is much smaller than those of other algorithms, and the results of multiple runs are highly consistent. Compared with the original NGO, INGO improves the accuracy by several orders of magnitude on functions such as F2, F3, F4, F15, and F16, verifying the effectiveness of the improved strategy. In summary, INGO has extremely high optimization accuracy, strong robustness, and fast convergence ability, making it an ideal algorithm for solving complex optimization problems. It presents lower optimization residuals than other comparative algorithms, which demonstrates that the improved algorithm maintains a reasonable balance between global exploration and local exploitation. For complex multimodal and discrete functions such as F6, F9, and F11, INGO can steadily approach the theoretical optimal value with small standard deviation, indicating that the improved strategies effectively avoid local extremum traps and enhance solution stability. In nonlinear optimization functions including F7, F10, F21, and F24, INGO presents smaller standard deviations and minor data fluctuations among repeated runs, which verifies its excellent robustness. Compared with the original NGO, INGO achieves multi-order-of-magnitude accuracy improvement on F2, F3, F4, F15, and F16, proving the positive optimization effect of chaotic initialization, nonlinear adaptive weight and Gaussian–Lévy mutation. The comprehensive experimental results show that INGO has competitive advantages in solving accuracy, stability, and convergence, and can effectively handle complex continuous optimization problems. In terms of nonlinear functions (F5, F7, F8, F10), INGO converges faster than other competitors, with no stagnation or rebound in the later iteration stage. In contrast, traditional algorithms such as BOA and GTO easily suffer from premature convergence, large curve fluctuation, and late-stage stagnation. Moreover, INGO can quickly obtain high-precision optimal solutions on F13, F17, F22, and F23, further verifying the effectiveness of the multi-strategy improvement mechanism. In general, INGO achieves comprehensive advantages in convergence speed, iterative stability, and solution accuracy, and it is suitable for nonlinear, complex and multi-extremum optimization scenarios.

According to the convergence curve analysis in Figure 2, INGO shows excellent convergence performance on all test functions. On high-dimensional complex functions such as F1, F2, and F3, the fitness value of INGO drops rapidly in the early iteration, usually approaching the theoretical optimal value within 100 generations, and the final accuracy generally reaches the order of $10^{-12}$ to $10^{-127}$, far better than comparison algorithms such as PSO, GWO, BOA, and GTO. On discrete or multimodal functions such as F6, F9, and F11, INGO can converge steadily to 0 with a smooth and oscillation-free convergence curve, showing a strong global optimization ability and the ability to avoid local optima. On functions such as F5, F7, F8, and F10, the convergence speed of INGO is significantly faster than those of other algorithms, and there is no rebound or stagnation in the later stage. In contrast, algorithms such as BOA and GTO often fall into local optima or converge slowly with large fluctuations. INGO also reaches machine precision at a very fast speed on functions such as F13, F17, F22, and F23, verifying the effectiveness of its improved strategy. In summary, INGO is significantly ahead of comparison algorithms in terms of convergence speed, solution accuracy, and stability, making it an ideal method for solving complex optimization problems.

## 6. Coverage Optimization of WSNs Based on Improved Northern Goshawk Algorithm

The improved Northern Goshawk Optimization (INGO) is applied to the coverage optimization of wireless sensor networks. The node deployment mathematical models of the 2D plane and 3D space are constructed respectively. With coverage rate, uniformity, and stability as the core evaluation indicators, multiple groups of comparative experiments are set up to comprehensively verify the algorithm performance. By comparing with PSO, GWO, BOA, GTO, DBO, SWO, and the original NGO, the ability of INGO to improve coverage, reduce coverage blind spots, and balance node distribution in complex spaces is mainly tested, and the practical application effect of the algorithm is comprehensively evaluated from the perspectives of convergence speed, optimization accuracy, and robustness, providing a simulation basis for verifying the effectiveness and engineering practicability of the improved strategy.

### 6.1. 2D Coverage Optimization of WSNs

To verify the effectiveness and superiority of the proposed INGO in the node deployment of wireless sensor networks (WSNs), a 2D coverage optimization simulation experiment is carried out based on the MATLAB 2023b platform. The experiment is set in a square monitoring area of $100\times 100\;m^{2}$, with N=40 sensor nodes randomly deployed and a sensing radius $R_{s}=10\;m$. The optimization goal is to maximize the regional coverage rate, with a grid precision of 50×50. The population size of all algorithms is uniformly set to 50, the maximum iteration number is set to 1000, and five independent runs are performed to eliminate randomness. Seven representative algorithms are selected as comparisons: Particle Swarm Optimization (PSO), the Grey Wolf Optimizer (GWO), the Butterfly Optimization Algorithm (BOA), Giraffe Toughening Optimization (GTO), the Dung Beetle Optimizer (DBO), Spider Wasp Optimization (SWO), and Northern Goshawk Optimization (NGO). The experiment records the convergence curve, average optimal coverage rate, and standard deviation of each algorithm. The results show that INGO has significant advantages in both convergence speed and final coverage rate, achieving a stable coverage improvement compared with NGO, verifying that the algorithm has stronger global optimization ability and engineering practical value in WSN deployment optimization. The optimized operation results of the eight algorithms are summarized in Table 5.

**Figure 3:**
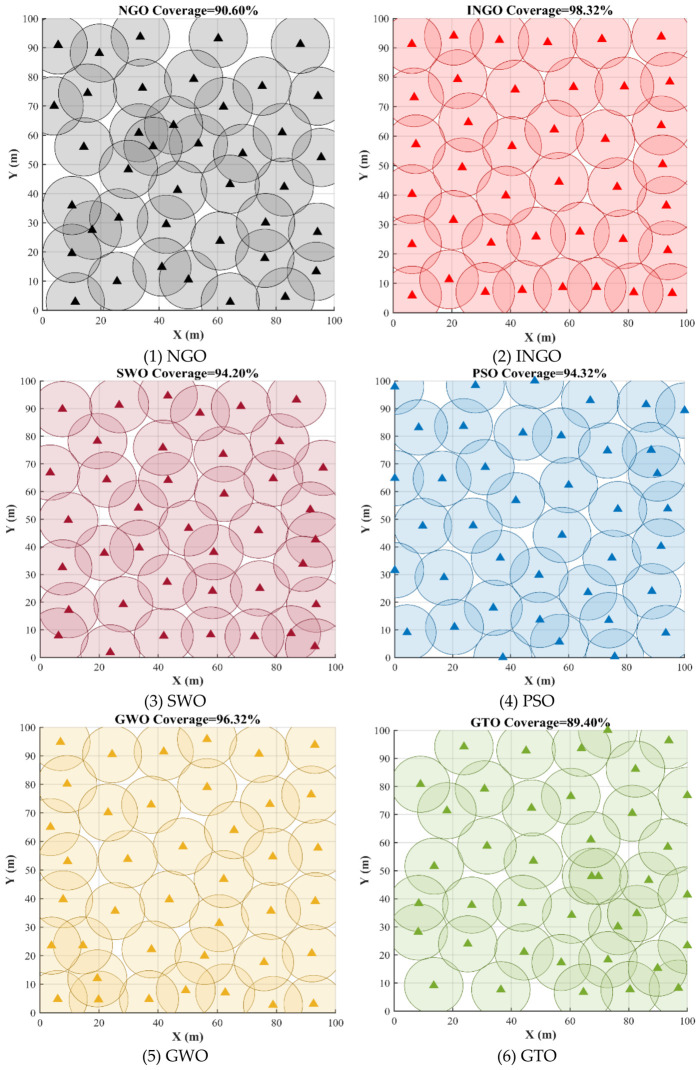

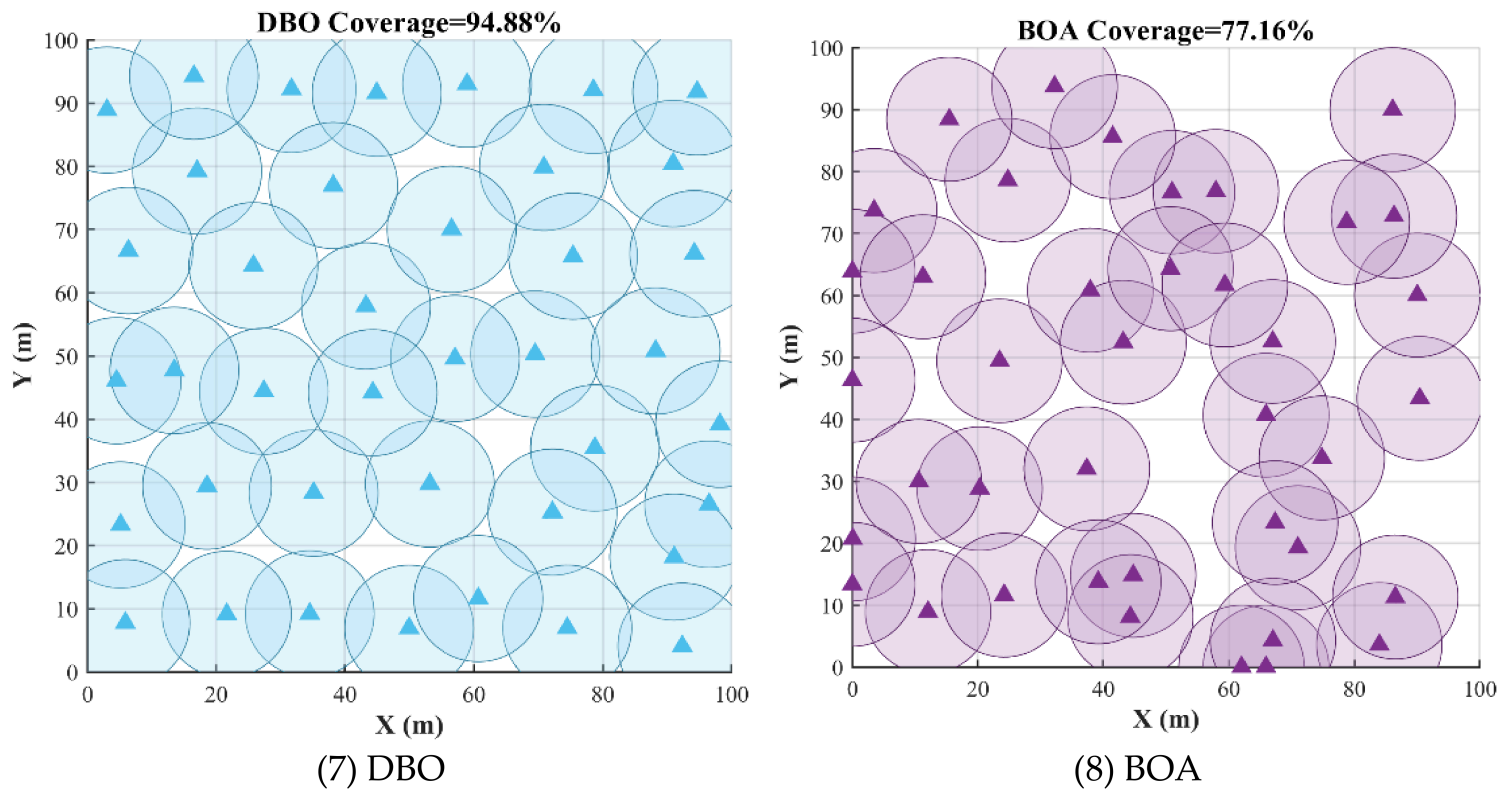
Comparison of coverage effects of two-dimensional wireless sensor networks.

Figure 3 shows the simulation graphs of the INGO algorithm, NGO algorithm, and six other comparative algorithms on the coverage optimization problem of two-dimensional wireless sensor networks.

The Improved Northern Goshawk Optimization (INGO) performs best, with a final coverage rate of 98.32% and a standard deviation of only 0.45%. The convergence curve shows that it reaches a stable high value within about 50 iterations, showing excellent global search ability and stability. Next are the improved or variants of the Grey Wolf Optimizer (GWO) and Butterfly Optimization Algorithm (BOA), but the basic version of BOA has a low coverage rate (about 77%). Particle Swarm Optimization (PSO), the Dung Beetle Optimizer (DBO), and Spider Wasp Optimization (SWO) have coverage rates concentrated in the range of 93–95%, with good performance but slightly inferior to INGO. From the node deployment diagram, INGO, PSO, and DBO have uniform layouts, effectively reducing coverage blind spots. In contrast, GWO, GTO, SWO, and NGO have obvious node aggregation along boundaries or grids, leading to internal coverage redundancy or insufficient edge coverage. In summary, INGO balances high coverage rate and high stability when solving WSN coverage deployment, and is the recommended optimization strategy.

### 6.2. 3D Coverage Optimization of WSNs

**Figure 4:**
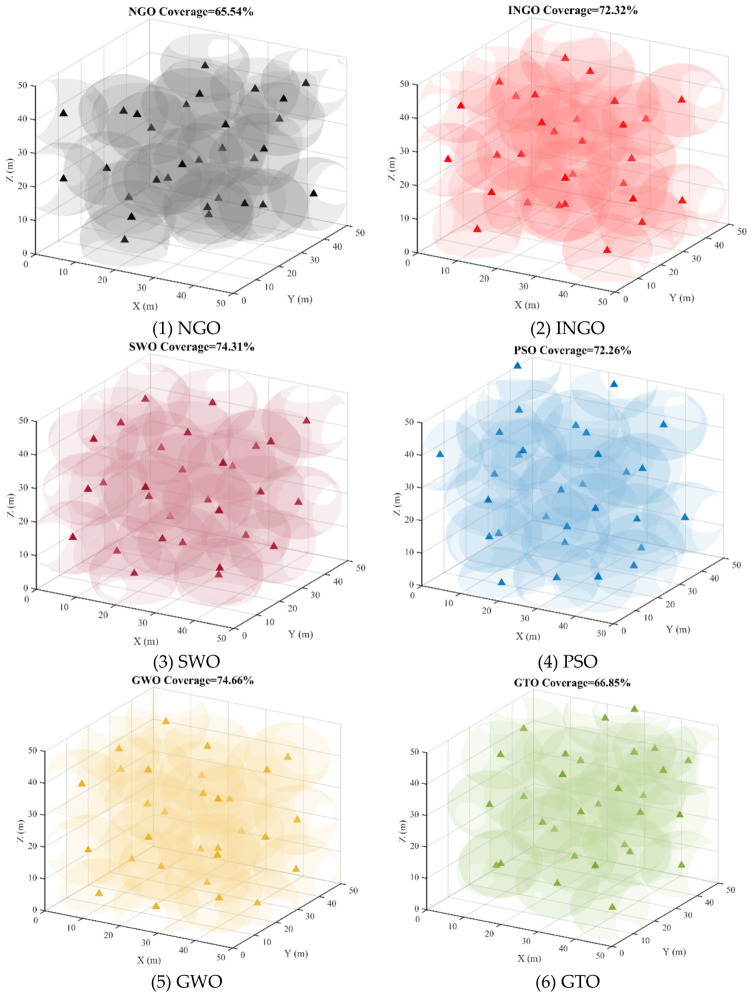

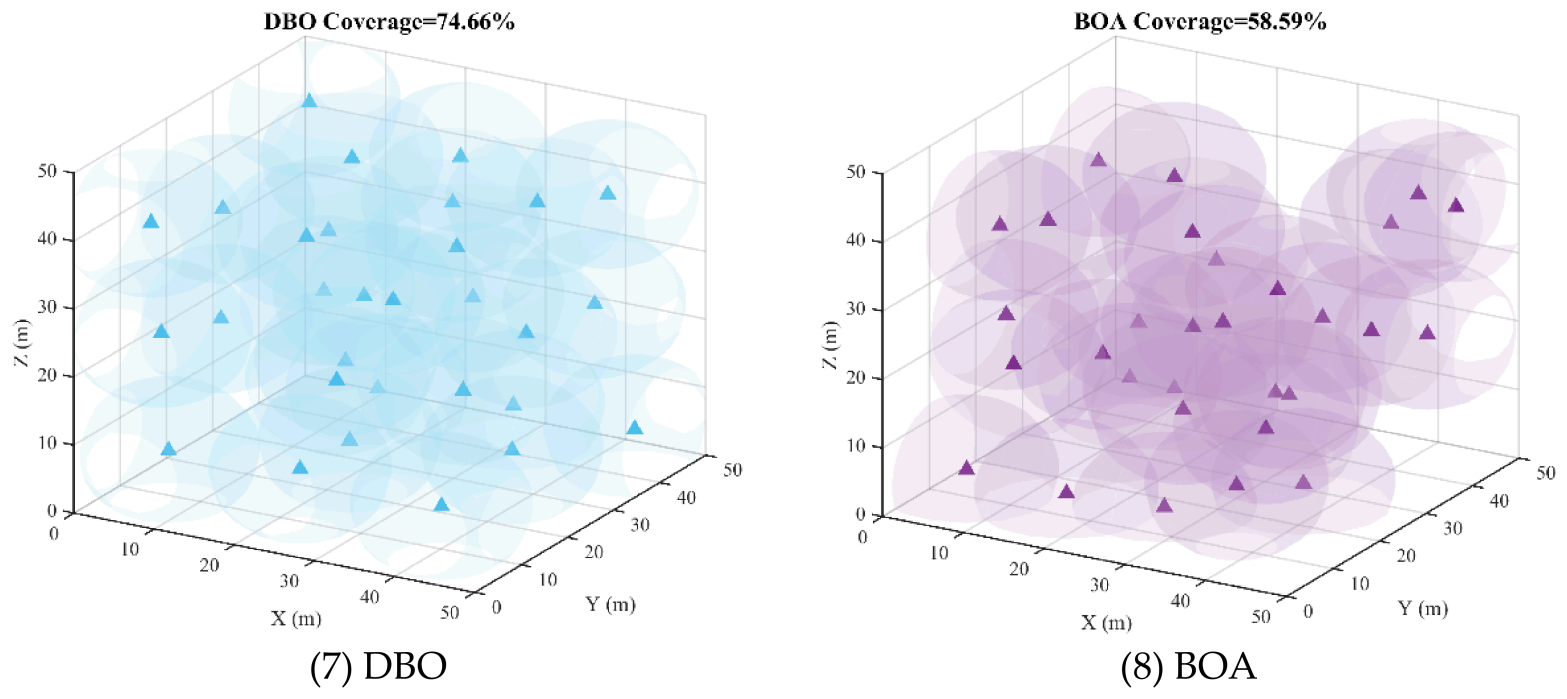
Comparison of coverage effects of 3D wireless sensor networks.

Based on the experimental results of 3D WSN coverage optimization, the Improved Northern Goshawk Optimization (INGO) shows significant superiority. In the deployment scenario of 30 sensor nodes with a sensing radius of 10 m in a $50\times 50\times 50{\;m}^{3}$ monitoring space, the best and average coverage rates of INGO reach 72.32% and 70.97%, respectively, which are 6.78 and 6.07 percentage points higher than the 65.54% and 64.90% of the original Northern Goshawk Optimization (NGO). This improvement shows that INGO effectively overcomes the deficiency of NGO in falling into local optima in 3D complex search space by introducing the improved mechanism, and significantly enhances the global exploration and deep exploitation abilities of the algorithm. Although the standard deviation (1.66%) of INGO is slightly higher than that of NGO (0.51%), it is still within the acceptable range of engineering, and the coverage accuracy is greatly improved at the cost of small stability. Compared with the other six comparison algorithms, INGO achieves the largest increase relative to NGO, fully verifying the pertinence and effectiveness of the improved strategy for 3D WSN node deployment. In summary, INGO has higher optimization accuracy and stronger spatial adaptability than the original NGO in 3D scenarios, showing excellent engineering application potential. The optimized operation results of the eight algorithms are summarized in Table 6. Figure 4 shows the simulation diagrams of INGO, NGO, and eight other comparison algorithms in 3D wireless sensor network coverage optimization.

Figure 4 shows the simulation diagrams of INGO, NGO, and other six comparison algorithms in 3D wireless sensor network coverage optimization. The Improved Northern Goshawk Optimization shows significant advantages in 3D WSN coverage optimization with outstanding comprehensive performance. Through strategies such as Tent chaotic initialization, adaptive weight, Gaussian–Lévy hybrid mutation, and Voronoi diagram guidance, the algorithm greatly improves the global search ability and optimization accuracy, effectively solving the problems of traditional algorithms such as tendency to fall into local optima, uneven node distribution, and many coverage blind spots. In 3D coverage simulation, the coverage rate of INGO reaches 72.32%, which is significantly improved compared with the original NGO, and also better than many comparison algorithms such as BOA and GTO, equivalent to mainstream algorithms such as PSO and GWO. It can be seen from the node distribution cloud diagram that INGO can achieve uniform deployment in the full space, with full coverage area, a reasonable node layout, a higher resource utilization rate, and stronger stability. Overall, INGO has stronger adaptability and robustness in complex 3D environments, and can efficiently improve network coverage, reduce coverage blind spots, and optimize node deployment effect, providing a more stable, efficient, and reliable solution for wireless sensor network coverage optimization, with important theoretical value and practical application prospects.

## 7. Conclusions

Aiming at the difficulties of wireless sensor network coverage optimization and the shortcomings of the basic Northern Goshawk Optimization algorithm, this paper proposes an Improved Northern Goshawk Optimization algorithm (INGO). The Tent chaotic map is used to optimize population initialization and improve the uniformity and ergodicity of initial solution distribution. Adaptive weight and a nonlinear learning factor are introduced to balance the global exploration and local exploitation abilities of the algorithm. Combined with Gaussian–Lévy hybrid mutation and Voronoi diagram guidance strategy, the ability of the algorithm to jump out of local optima is enhanced, and the uniform deployment of sensor nodes is realized. Simulation experiments are carried out through CEC2017 standard test functions and 2D/3D sensor network coverage scenarios. From the perspective of engineering application, the designed INGO has a lightweight structure and stable convergence performance. It can effectively solve the node deployment problem of two-dimensional and three-dimensional wireless sensor networks. By optimizing spatial node distribution and reducing coverage overlap and blind areas, the algorithm can provide reliable deployment solutions for intelligent sensing monitoring, industrial environment perception, and field regional monitoring, which confirms its practical engineering value. Meanwhile, the inherent limitations of INGO are objectively summarized. Firstly, all simulation experiments are carried out under ideal obstacle-free environments without considering complex terrain, communication interference, and node failure. Secondly, the multi-strategy fusion mechanism brings slight computational overhead compared with the original NGO. Thirdly, all parameters adopt fixed typical values without extensive sensitivity traversal tests.

Future research can be further carried out in terms of algorithm optimization and engineering application. At the algorithm level, mechanisms such as adaptive restart and multi-strategy collaborative search can be integrated to improve the adaptability of the algorithm in ultra-high-dimensional, strongly constrained, and dynamically changing scenarios. At the same time, an integrated multi-objective optimization model of coverage, energy consumption, delay, and connectivity can be constructed to meet more complex engineering requirements. At the application level, INGO can be combined with edge computing and IoT terminals to achieve lightweight and low-latency real-time deployment. The algorithm can be extended to dynamic scenarios such as mobile sensor networks, UAV cooperative monitoring, and smart mines. In addition, hardware-in-the-loop experiments and actual site deployment tests can be carried out to improve the engineering adaptation process of the algorithm, promote the improved algorithm from theoretical simulation to practical application, and provide more efficient and stable optimization support for smart perception, environmental monitoring, the industrial Internet of Things, and other fields.

## Figures and Tables

**Table 1:** CEC2017 benchmark test set.

Function Type	Function	Name	Theoretical Optimum
Unimodal function	F1	Shifted and Rotated Bent Cigar Function	100
	F2	Shifted and Rotated Sum of Different Power Function	200
	F3	Shifted and Rotated Zakharov Function	300
Simple multimodal function	F4	Shifted and Rotated Rosenbrock’s Function	400
	F5	Shifted and Rotated Rastrigin’s Function	500
	F6	Shifted and Rotated Expanded Scaffer’s F6 Function	600
	F7	Shifted and Rotated Lunacek Bi_Rastrigin Function	700
	F8	Rotated Non-Continuous Rastrigin’s Function	800
	F9	Shifted and Rotated Levy Function	900
	F10	Shifted and Rotated Schwefel’s Function	1000
Mixed function	F11	Hybrid Function 1 (N = 3)	1100
	F12	Hybrid Function 2 (N = 3)	1200
	F13	Hybrid Function 3 (N = 3)	1300
	F14	Hybrid Function 4 (N = 4)	1400
	F15	Hybrid Function 5 (N = 4)	1500
	F16	Hybrid Function 6 (N = 4)	1600
	F17	Hybrid Function 6 (N = 5)	1700
	F18	Hybrid Function 6 (N = 5)	1800
	F19	Hybrid Function 6 (N = 5)	1900
	F20	Hybrid Function 6 (N = 6)	2000
Composite function	F21	Composition Function 1	2100
	F22	Composition Function 2	2200
	F23	Composition Function 3	2300
	F24	Composition Function 4	2400
	F25	Composition Function 5	2500
	F26	Composition Function 6	2600
	F27	Composition Function 7	2700
	F28	Composition Function 8	2800
	F29	Composition Function 9	2900
	F30	Composition Function 10	3000

**Table 2:** Results of different swarm intelligence algorithms (Dim = 30).

Function	Metric	PSO	GWO	BOA	GTO	DBO	SWO	NGO	INGO
F1	Best	1.65 × 10^−1^	3.03 × 10^−32^	8.76 × 10^−2^	1.69 × 10^3^	7.13 × 10^0^	5.12 × 10^0^	3.19 × 10^−94^	5.78 × 10^−127^
F1	Avg	4.79 × 10^−1^	1.95 × 10^−31^	9.27 × 10^−2^	3.72 × 10^3^	1.39 × 10^1^	7.35 × 10^0^	6.45 × 10^−92^	4.76 × 10^−125^
F1	Std	2.91 × 10^−1^	1.31 × 10^−31^	3.21 × 10^−3^	1.60 × 10^3^	4.28 × 10^0^	1.87 × 10^0^	1.42 × 10^−91^	8.47 × 10^−125^
F2	Best	5.27 × 10^0^	5.29 × 10^−18^	4.31 × 10^38^	1.57 × 10^9^	2.78 × 10^3^	8.14 × 10^0^	9.50 × 10^−48^	6.37 × 10^−64^
F2	Avg	1.07 × 10^2^	1.43 × 10^−17^	1.35 × 10^44^	6.62 × 10^20^	9.07 × 10^18^	3.33 × 10^1^	1.07 × 10^−46^	4.59 × 10^−63^
F2	Std	1.14 × 10^2^	5.96 × 10^−18^	4.20 × 10^44^	2.09 × 10^21^	2.58 × 10^19^	1.41 × 10^1^	9.40 × 10^−47^	4.26 × 10^−63^
F3	Best	1.42 × 10^3^	3.21 × 10^−7^	9.24 × 10^−2^	4.31 × 10^3^	1.60 × 10^3^	5.84 × 10^2^	7.50 × 10^−27^	1.11 × 10^−56^
F3	Avg	3.96 × 10^3^	2.67 × 10^−5^	9.79 × 10^−2^	1.22 × 10^4^	3.39 × 10^3^	1.09 × 10^3^	9.34 × 10^−24^	1.19 × 10^−47^
F3	Std	2.82 × 10^3^	2.20 × 10^−5^	4.93 × 10^−3^	5.62 × 10^3^	1.18 × 10^3^	3.95 × 10^2^	2.00 × 10^−23^	2.85 × 10^−47^
F4	Best	5.42 × 10^0^	3.26 × 10^−7^	4.08 × 10^−1^	3.83 × 10^1^	4.80 × 10^0^	4.78 × 10^0^	9.48 × 10^−40^	3.72 × 10^−55^
F4	Avg	8.63 × 10^0^	4.82 × 10^−6^	4.42 × 10^−1^	4.28 × 10^1^	1.11 × 10^1^	8.29 × 10^0^	8.96 × 10^−39^	3.14 × 10^−54^
F4	Std	1.52 × 10^0^	6.19 × 10^−6^	1.63 × 10^−2^	3.25 × 10^0^	5.38 × 10^0^	2.71 × 10^0^	1.00 × 10^−38^	3.53 × 10^−54^
F5	Best	7.61 × 10^2^	2.58 × 10^1^	2.89 × 10^1^	5.85 × 10^7^	5.76 × 10^3^	3.39 × 10^3^	2.52 × 10^1^	2.38 × 10^1^
F5	Avg	1.03 × 10^5^	2.69 × 10^1^	2.89 × 10^1^	1.80 × 10^8^	6.64 × 10^4^	1.15 × 10^4^	2.58 × 10^1^	2.45 × 10^1^
F5	Std	3.16 × 10^5^	1.13 × 10^0^	2.34 × 10^−2^	1.08 × 10^8^	6.95 × 10^4^	6.22 × 10^3^	3.37 × 10^−1^	5.50 × 10^−1^
F6	Best	2.00 × 10^0^	0.00 × 10^0^	5.10 × 10^1^	1.48 × 10^3^	1.00 × 10^1^	9.00 × 10^0^	0.00 × 10^0^	0.00 × 10^0^
F6	Avg	4.00 × 10^0^	0.00 × 10^0^	6.07 × 10^1^	2.69 × 10^3^	2.16 × 10^1^	1.73 × 10^1^	0.00 × 10^0^	0.00 × 10^0^
F6	Std	1.83 × 10^0^	0.00 × 10^0^	5.85 × 10^0^	1.16 × 10^3^	1.04 × 10^1^	6.07 × 10^0^	0.00 × 10^0^	0.00 × 10^0^
F7	Best	9.91 × 10^0^	2.13 × 10^−3^	7.26 × 10^−1^	5.87 × 10^6^	5.32 × 10^2^	1.17 × 10^2^	2.27 × 10^−4^	6.81 × 10^−5^
F7	Avg	2.54 × 10^2^	4.32 × 10^−3^	7.80 × 10^−1^	3.60 × 10^7^	1.74 × 10^3^	5.56 × 10^2^	5.48 × 10^−4^	1.86 × 10^−4^
F7	Std	3.48 × 10^2^	1.72 × 10^−3^	5.53 × 10^−2^	1.83 × 10^7^	1.10 × 10^3^	3.81 × 10^2^	2.30 × 10^−4^	1.04 × 10^−4^
F8	Best	−1.60 × 10^3^	−1.35 × 10^3^	−1.02 × 10^3^	−1.55 × 10^3^	−1.67 × 10^3^	−1.75 × 10^3^	−1.57 × 10^3^	−1.75 × 10^3^
F8	Avg	−1.48 × 10^3^	−1.18 × 10^3^	−9.15 × 10^2^	−1.35 × 10^3^	−1.50 × 10^3^	−1.66 × 10^3^	−1.46 × 10^3^	−1.56 × 10^3^
F8	Std	9.54 × 10^1^	1.56 × 10^2^	5.61 × 10^1^	1.15 × 10^2^	9.98 × 10^1^	9.48 × 10^1^	9.65 × 10^1^	1.17 × 10^2^
F9	Best	8.76 × 10^1^	5.68 × 10^−14^	1.92 × 10^2^	1.53 × 10^3^	2.57 × 10^2^	2.39 × 10^2^	0.00 × 10^0^	0.00 × 10^0^
F9	Avg	1.47 × 10^2^	8.50 × 10^0^	2.27 × 10^2^	3.05 × 10^3^	3.10 × 10^2^	3.97 × 10^2^	0.00 × 10^0^	1.60 × 10^0^
F9	Std	3.25 × 10^1^	6.27 × 10^0^	2.33 × 10^1^	9.09 × 10^2^	5.24 × 10^1^	9.68 × 10^1^	0.00 × 10^0^	5.05 × 10^0^
F10	Best	2.00 × 10^1^	2.06 × 10^1^	2.09 × 10^1^	2.00 × 10^1^	2.00 × 10^1^	4.55 × 10^0^	3.42 × 10^−13^	2.00 × 10^1^
F10	Avg	2.02 × 10^1^	2.09 × 10^1^	2.11 × 10^1^	2.00 × 10^1^	2.00 × 10^1^	1.85 × 10^1^	1.60 × 10^1^	2.00 × 10^1^
F10	Std	2.49 × 10^−1^	1.14 × 10^−1^	6.43 × 10^−2^	0.00 × 10^0^	4.03 × 10^−7^	4.89 × 10^0^	8.37 × 10^0^	5.68 × 10^−10^
F11	Best	2.14 × 10^−2^	0.00 × 10^0^	4.42 × 10^−1^	1.32 × 10^0^	3.63 × 10^−1^	2.83 × 10^−1^	0.00 × 10^0^	0.00 × 10^0^
F11	Avg	6.70 × 10^−2^	5.73 × 10^−3^	4.79 × 10^−1^	2.00 × 10^0^	5.95 × 10^−1^	3.91 × 10^−1^	0.00 × 10^0^	0.00 × 10^0^
F11	Std	7.36 × 10^−2^	7.71 × 10^−3^	3.31 × 10^−2^	3.65 × 10^−1^	1.25 × 10^−1^	7.08 × 10^−2^	0.00 × 10^0^	0.00 × 10^0^
F12	Best	1.16 × 10^2^	2.02 × 10^−27^	1.06 × 10^0^	3.23 × 10^3^	2.51 × 10^2^	3.12 × 10^2^	6.33 × 10^−92^	6.57 × 10^−127^
F12	Avg	1.51 × 10^2^	4.97 × 10^0^	1.29 × 10^2^	7.67 × 10^3^	3.35 × 10^2^	4.01 × 10^2^	7.62 × 10^−91^	3.35 × 10^−122^
F12	Std	3.69 × 10^1^	6.68 × 10^0^	7.26 × 10^1^	2.43 × 10^3^	6.22 × 10^1^	7.68 × 10^1^	1.35 × 10^−90^	7.85 × 10^−122^
F13	Best	1.90 × 10^3^	2.76 × 10^−9^	2.46 × 10^−1^	6.63 × 10^3^	2.51 × 10^3^	5.86 × 10^2^	4.44 × 10^−16^	4.44 × 10^−16^
F13	Avg	4.31 × 10^3^	2.43 × 10^−5^	2.70 × 10^−1^	1.33 × 10^4^	3.01 × 10^3^	1.14 × 10^3^	4.44 × 10^−16^	4.44 × 10^−16^
F13	Std	3.11 × 10^3^	7.24 × 10^−5^	2.42 × 10^−2^	6.95 × 10^3^	5.42 × 10^2^	3.32 × 10^2^	3.29 × 10^−32^	0.00 × 10^0^
F14	Best	6.63 × 10^2^	2.60 × 10^1^	2.89 × 10^1^	4.09 × 10^7^	6.00 × 10^3^	2.00 × 10^3^	2.61 × 10^1^	2.48 × 10^1^
F14	Avg	4.64 × 10^3^	2.70 × 10^1^	2.89 × 10^1^	2.13 × 10^8^	3.14 × 10^4^	6.57 × 10^3^	2.65 × 10^1^	2.57 × 10^1^
F14	Std	4.32 × 10^3^	6.64 × 10^−1^	3.62 × 10^−2^	1.96 × 10^8^	5.83 × 10^4^	5.03 × 10^3^	3.06 × 10^−1^	5.78 × 10^−1^
F15	Best	8.67 × 10^−1^	4.33 × 10^−2^	2.43 × 10^39^	2.72 × 10^8^	3.24 × 10^4^	1.99 × 10^0^	1.88 × 10^−47^	2.52 × 10^−64^
F15	Avg	8.09 × 10^1^	6.93 × 10^−1^	2.37 × 10^42^	1.05 × 10^23^	6.84 × 10^18^	2.59 × 10^1^	3.33 × 10^−46^	2.49 × 10^−4^
F15	Std	8.22 × 10^1^	6.13 × 10^−1^	4.31 × 10^42^	3.32 × 10^23^	2.05 × 10^19^	2.37 × 10^1^	3.83 × 10^−46^	7.88 × 10^−4^
F16	Best	6.85 × 10^1^	4.76 × 10^0^	2.10 × 10^2^	2.27 × 10^3^	2.61 × 10^2^	2.55 × 10^2^	2.59 × 10^−40^	1.33 × 10^−54^
F16	Avg	1.18 × 10^2^	1.17 × 10^1^	2.40 × 10^2^	4.63 × 10^3^	3.33 × 10^2^	4.23 × 10^2^	2.48 × 10^−39^	6.40 × 10^0^
F16	Std	3.76 × 10^1^	7.85 × 10^0^	2.04 × 10^1^	1.92 × 10^3^	5.67 × 10^1^	1.02 × 10^2^	1.82 × 10^−39^	1.05 × 10^1^
F17	Best	9.52 × 10^−1^	4.00 × 10^−15^	2.39 × 10^−1^	1.19 × 10^3^	1.14 × 10^1^	9.07 × 10^0^	4.44 × 10^−16^	4.44 × 10^−16^
F17	Avg	1.84 × 10^0^	4.00 × 10^−15^	2.50 × 10^−1^	2.94 × 10^3^	2.02 × 10^1^	1.17 × 10^1^	4.44 × 10^−16^	4.44 × 10^−16^
F17	Std	1.17 × 10^0^	1.86 × 10^−30^	6.59 × 10^−3^	1.29 × 10^3^	7.83 × 10^0^	2.21 × 10^0^	7.35 × 10^−32^	0.00 × 10^0^
F18	Best	1.36 × 10^3^	4.25 × 10^−7^	8.49 × 10^−2^	6.40 × 10^3^	1.57 × 10^3^	2.49 × 10^2^	3.94 × 10^−31^	1.17 × 10^−55^
F18	Avg	3.83 × 10^3^	2.26 × 10^−4^	1.01 × 10^−1^	1.01 × 10^4^	3.27 × 10^3^	1.23 × 10^3^	2.76 × 10^−25^	7.18 × 10^−51^
F18	Std	2.65 × 10^3^	6.79 × 10^−4^	8.51 × 10^−3^	2.83 × 10^3^	1.19 × 10^3^	5.96 × 10^2^	7.82 × 10^−25^	1.61 × 10^−50^
F19	Best	4.06 × 10^2^	2.45 × 10^1^	2.89 × 10^1^	8.36 × 10^7^	6.47 × 10^3^	2.56 × 10^3^	2.13 × 10^1^	1.85 × 10^1^
F19	Avg	2.02 × 10^5^	2.62 × 10^1^	2.89 × 10^1^	2.74 × 10^8^	5.68 × 10^4^	8.94 × 10^3^	2.30 × 10^1^	2.00 × 10^1^
F19	Std	4.21 × 10^5^	1.19 × 10^0^	3.55 × 10^−2^	2.64 × 10^8^	8.06 × 10^4^	5.49 × 10^3^	1.04 × 10^0^	1.10 × 10^0^
F20	Best	3.81 × 10^2^	1.44 × 10^−15^	5.18 × 10^39^	3.35 × 10^9^	1.24 × 10^5^	3.29 × 10^2^	6.99 × 10^−47^	1.56 × 10^−63^
F20	Avg	1.13 × 10^4^	5.88 × 10^0^	1.08 × 10^43^	1.23 × 10^18^	5.17 × 10^21^	6.67 × 10^2^	7.38 × 10^−46^	2.61 × 10^−62^
F20	Std	7.42 × 10^3^	6.40 × 10^0^	1.67 × 10^43^	2.77 × 10^18^	1.64 × 10^22^	6.42 × 10^2^	7.92 × 10^−46^	3.20 × 10^−62^
F21	Best	5.77 × 10^2^	2.89 × 10^1^	2.90 × 10^1^	4.11 × 10^7^	9.30 × 10^3^	5.06 × 10^3^	2.89 × 10^1^	2.89 × 10^1^
F21	Avg	1.05 × 10^5^	2.89 × 10^1^	2.90 × 10^1^	3.13 × 10^8^	4.20 × 10^4^	1.59 × 10^4^	2.89 × 10^1^	2.89 × 10^1^
F21	Std	3.22 × 10^5^	5.11 × 10^−3^	1.25 × 10^−2^	3.20 × 10^8^	4.56 × 10^4^	1.01 × 10^4^	5.19 × 10^−6^	3.00 × 10^−7^
F22	Best	2.83 × 10^2^	2.01 × 10^−15^	5.64 × 10^39^	5.50 × 10^11^	8.69 × 10^8^	1.25 × 10^2^	4.44 × 10^−16^	4.44 × 10^−16^
F22	Avg	8.10 × 10^3^	2.54 × 10^−14^	2.89 × 10^43^	4.44 × 10^19^	4.34 × 10^17^	1.14 × 10^3^	4.44 × 10^−16^	4.44 × 10^−16^
F22	Std	8.09 × 10^3^	4.46 × 10^−14^	5.04 × 10^43^	9.34 × 10^19^	9.55 × 10^17^	1.06 × 10^3^	6.57 × 10^−32^	0.00 × 10^0^
F23	Best	3.60 × 10^−1^	4.00 × 10^−15^	2.33 × 10^−1^	1.93 × 10^3^	9.36 × 10^0^	8.34 × 10^0^	4.44 × 10^−16^	4.44 × 10^−16^
F23	Avg	1.46 × 10^0^	4.00 × 10^−15^	2.47 × 10^−1^	3.36 × 10^3^	1.61 × 10^1^	1.20 × 10^1^	4.44 × 10^−16^	4.44 × 10^−16^
F23	Std	6.67 × 10^−1^	1.88 × 10^−30^	8.26 × 10^−3^	1.91 × 10^3^	3.49 × 10^0^	2.56 × 10^0^	5.69 × 10^−32^	0.00 × 10^0^
F24	Best	2.49 × 10^3^	2.90 × 10^1^	2.90 × 10^1^	7.45 × 10^7^	4.96 × 10^3^	3.84 × 10^3^	2.89 × 10^1^	2.89 × 10^1^
F24	Avg	1.11 × 10^5^	2.90 × 10^1^	2.90 × 10^1^	3.56 × 10^8^	4.52 × 10^4^	1.19 × 10^4^	2.89 × 10^1^	2.89 × 10^1^
F24	Std	3.20 × 10^5^	1.91 × 10^−3^	7.19 × 10^−3^	2.13 × 10^8^	5.38 × 10^4^	6.93 × 10^3^	5.88 × 10^−6^	1.23 × 10^−4^
F25	Best	1.39 × 10^2^	1.16 × 10^−13^	3.75 × 10^38^	1.04 × 10^12^	3.57 × 10^3^	1.89 × 10^2^	5.73 × 10^−47^	1.46 × 10^−63^
F25	Avg	9.35 × 10^3^	8.26 × 10^0^	4.12 × 10^43^	4.78 × 10^18^	2.87 × 10^20^	4.75 × 10^2^	2.92 × 10^−46^	2.35 × 10^−62^
F25	Std	7.51 × 10^3^	8.25 × 10^0^	9.04 × 10^43^	1.41 × 10^19^	9.08 × 10^20^	2.60 × 10^2^	2.36 × 10^−46^	3.03 × 10^−62^
F26	Best	1.46 × 10^1^	4.50 × 10^−16^	6.87 × 10^39^	5.28 × 10^13^	3.09 × 10^3^	1.72 × 10^1^	4.44 × 10^−16^	4.44 × 10^−16^
F26	Avg	8.17 × 10^3^	4.93 × 10^−16^	1.02 × 10^43^	3.12 × 10^20^	6.60 × 10^17^	1.52 × 10^2^	4.44 × 10^−16^	4.44 × 10^−16^
F26	Std	6.40 × 10^3^	5.31 × 10^−17^	2.21 × 10^43^	9.63 × 10^20^	1.41 × 10^18^	2.90 × 10^2^	3.29 × 10^−32^	0.00 × 10^0^
F27	Best	1.61 × 10^3^	−1.71 × 10^−1^	7.86 × 10^−2^	6.77 × 10^3^	1.70 × 10^3^	6.77 × 10^2^	−2.17 × 10^−1^	−2.37 × 10^−1^
F27	Avg	2.93 × 10^3^	−9.73 × 10^−2^	9.26 × 10^−2^	1.17 × 10^4^	2.89 × 10^3^	1.11 × 10^3^	−1.84 × 10^−1^	−1.82 × 10^−1^
F27	Std	1.56 × 10^3^	5.51 × 10^−2^	1.02 × 10^−2^	3.39 × 10^3^	8.63 × 10^2^	3.22 × 10^2^	4.66 × 10^−2^	5.39 × 10^−2^
F28	Best	3.52 × 10^2^	2.84 × 10^1^	2.89 × 10^1^	9.43 × 10^7^	6.04 × 10^3^	3.52 × 10^3^	2.84 × 10^1^	2.83 × 10^1^
F28	Avg	1.03 × 10^5^	2.85 × 10^1^	2.90 × 10^1^	2.23 × 10^8^	3.59 × 10^4^	1.79 × 10^4^	2.84 × 10^1^	2.84 × 10^1^
F28	Std	3.19 × 10^5^	6.52 × 10^−2^	3.19 × 10^−2^	1.55 × 10^8^	3.82 × 10^4^	2.62 × 10^4^	1.54 × 10^−2^	4.78 × 10^−3^
F29	Best	6.75 × 10^2^	1.29 × 10^−14^	4.14 × 10^39^	2.43 × 10^15^	1.28 × 10^6^	8.99 × 10^2^	1.04 × 10^−46^	1.97 × 10^−63^
F29	Avg	2.08 × 10^4^	1.72 × 10^1^	2.27 × 10^42^	2.72 × 10^19^	7.98 × 10^20^	3.31 × 10^3^	5.53 × 10^−46^	2.12 × 10^−62^
F29	Std	2.05 × 10^4^	1.69 × 10^1^	5.74 × 10^42^	6.61 × 10^19^	1.31 × 10^21^	2.96 × 10^3^	4.33 × 10^−46^	3.34 × 10^−62^
F30	Best	3.72 × 10^2^	2.86 × 10^1^	2.89 × 10^1^	7.52 × 10^7^	4.54 × 10^3^	1.83 × 10^3^	2.85 × 10^1^	2.85 × 10^1^
F30	Avg	5.16 × 10^3^	2.86 × 10^1^	2.90 × 10^1^	3.29 × 10^8^	9.39 × 10^3^	9.01 × 10^3^	2.85 × 10^1^	2.85 × 10^1^
F30	Std	6.90 × 10^3^	6.22 × 10^−2^	2.20 × 10^−2^	1.38 × 10^8^	5.81 × 10^3^	1.11 × 10^4^	1.45 × 10^−3^	3.28 × 10^−3^

**Table 3:** Solution results obtained from different algorithms (Dim = 50).

Function	Metric	PSO	GWO	BOA	GTO	DBO	SWO	NGO	INGO
F1	Best	3.13 × 10^1^	2.46 × 10^−23^	9.54 × 10^−2^	9.08 × 10^3^	7.15 × 10^1^	5.20 × 10^1^	6.26 × 10^−91^	1.74 × 10^−124^
F1	Avg	1.06 × 10^3^	2.18 × 10^−22^	1.02 × 10^−1^	1.33 × 10^4^	1.05 × 10^2^	6.74 × 10^1^	6.17 × 10^−90^	4.11 × 10^−120^
F1	Std	3.15 × 10^3^	2.13 × 10^−22^	3.21 × 10^−3^	3.27 × 10^3^	1.82 × 10^1^	1.36 × 10^1^	1.19 × 10^−89^	1.29 × 10^−119^
F2	Best	3.94 × 10^2^	3.41 × 10^−13^	9.87 × 10^65^	1.29 × 10^32^	1.51 × 10^22^	1.51 × 10^2^	6.61 × 10^−47^	2.00 × 10^−62^
F2	Avg	3.19 × 10^18^	1.29 × 10^−12^	4.73 × 10^73^	2.74 × 10^42^	1.32 × 10^43^	3.43 × 10^2^	1.14 × 10^−45^	1.56 × 10^−61^
F2	Std	1.01 × 10^19^	7.65 × 10^−13^	1.45 × 10^74^	3.70 × 10^42^	4.16 × 10^43^	1.49 × 10^2^	1.06 × 10^−45^	2.32 × 10^−61^
F3	Best	1.25 × 10^4^	3.38 × 10^−1^	9.91 × 10^−2^	2.87 × 10^4^	6.09 × 10^3^	3.61 × 10^3^	2.79 × 10^−24^	7.95 × 10^−48^
F3	Avg	2.59 × 10^4^	7.26 × 10^0^	1.10 × 10^−1^	3.98 × 10^4^	1.55 × 10^4^	5.20 × 10^3^	2.92 × 10^−19^	2.43 × 10^−41^
F3	Std	9.42 × 10^3^	7.99 × 10^0^	8.12 × 10^−3^	8.73 × 10^3^	4.37 × 10^3^	1.16 × 10^3^	8.54 × 10^−19^	7.65 × 10^−41^
F4	Best	1.60 × 10^1^	1.06 × 10^−3^	4.59 × 10^−1^	4.10 × 10^1^	1.34 × 10^1^	1.41 × 10^1^	2.05 × 10^−38^	5.36 × 10^−53^
F4	Avg	2.04 × 10^1^	4.37 × 10^−3^	4.88 × 10^−1^	5.18 × 10^1^	2.17 × 10^1^	1.75 × 10^1^	3.45 × 10^−37^	4.79 × 10^−52^
F4	Std	4.09 × 10^0^	2.78 × 10^−3^	2.06 × 10^−2^	7.89 × 10^0^	3.48 × 10^0^	2.65 × 10^0^	2.75 × 10^−37^	3.99 × 10^−52^
F5	Best	9.14 × 10^4^	4.60 × 10^1^	4.89 × 10^1^	8.56 × 10^8^	9.12 × 10^4^	9.38 × 10^4^	4.58 × 10^1^	4.48 × 10^1^
F5	Avg	3.76 × 10^5^	4.73 × 10^1^	4.89 × 10^1^	1.91 × 10^9^	3.01 × 10^5^	3.12 × 10^5^	4.66 × 10^1^	4.59 × 10^1^
F5	Std	3.95 × 10^5^	1.14 × 10^0^	2.31 × 10^−2^	1.02 × 10^9^	2.47 × 10^5^	1.57 × 10^5^	6.55 × 10^−1^	1.11 × 10^0^
F6	Best	6.30 × 10^1^	0.00 × 10^0^	4.10 × 10^1^	6.86 × 10^3^	9.30 × 10^1^	6.70 × 10^1^	0.00 × 10^0^	0.00 × 10^0^
F6	Avg	1.12 × 10^2^	0.00 × 10^0^	5.06 × 10^1^	1.17 × 10^4^	1.30 × 10^2^	1.15 × 10^2^	3.00 × 10^−1^	0.00 × 10^0^
F6	Std	3.03 × 10^1^	0.00 × 10^0^	6.10 × 10^0^	3.37 × 10^3^	2.70 × 10^1^	2.18 × 10^1^	4.83 × 10^−1^	0.00 × 10^0^
F7	Best	3.16 × 10^4^	3.97 × 10^−3^	7.34 × 10^−1^	8.90 × 10^7^	5.71 × 10^4^	1.55 × 10^4^	7.57 × 10^−5^	3.11 × 10^−5^
F7	Avg	2.01 × 10^7^	6.50 × 10^−3^	7.95 × 10^−1^	4.37 × 10^8^	1.47 × 10^5^	7.61 × 10^4^	5.89 × 10^−4^	2.40 × 10^−4^
F7	Std	6.32 × 10^7^	2.00 × 10^−3^	3.43 × 10^−2^	1.86 × 10^8^	6.51 × 10^4^	4.96 × 10^4^	3.33 × 10^−4^	1.72 × 10^−4^
F8	Best	−2.65 × 10^3^	−2.08 × 10^3^	−1.51 × 10^3^	−2.10 × 10^3^	−2.51 × 10^3^	−2.79 × 10^3^	−2.45 × 10^3^	2.91 × 10^3^
F8	Avg	2.47 × 10^3^	1.77 × 10^3^	1.23 × 10^3^	1.85 × 10^3^	2.42 × 10^3^	2.64 × 10^3^	2.08 × 10^3^	2.59 × 10^3^
F8	Std	1.33 × 10^2^	1.92 × 10^2^	1.20 × 10^2^	1.66 × 10^2^	8.29 × 10^1^	1.97 × 10^2^	2.11 × 10^2^	1.74 × 10^2^
F9	Best	4.24 × 10^2^	1.07 × 10^1^	3.29 × 10^2^	9.86 × 10^3^	6.63 × 10^2^	5.85 × 10^2^	0.00 × 10^0^	0.00 × 10^0^
F9	Avg	5.29 × 10^2^	2.46 × 10^1^	3.54 × 10^2^	1.50 × 10^4^	7.96 × 10^2^	8.44 × 10^2^	0.00 × 10^0^	0.00 × 10^0^
F9	Std	7.51 × 10^1^	9.08 × 10^0^	1.90 × 10^1^	3.01 × 10^3^	9.96 × 10^1^	3.42 × 10^2^	0.00 × 10^0^	0.00 × 10^0^
F10	Best	2.00 × 10^1^	2.09 × 10^1^	2.12 × 10^1^	2.00 × 10^1^	2.00 × 10^1^	2.00 × 10^1^	1.80 × 10^0^	2.00 × 10^1^
F10	Avg	2.03 × 10^1^	2.11 × 10^1^	2.13 × 10^1^	2.00 × 10^1^	2.00 × 10^1^	2.00 × 10^1^	1.82 × 10^1^	2.00 × 10^1^
F10	Std	4.32 × 10^−1^	7.30 × 10^−2^	3.67 × 10^−2^	0.00 × 10^0^	4.44 × 10^−3^	0.00 × 10^0^	5.76 × 10^0^	0.00 × 10^0^
F11	Best	6.26 × 10^−1^	0.00 × 10^0^	4.43 × 10^−1^	3.61 × 10^0^	8.89 × 10^−1^	7.30 × 10^−1^	0.00 × 10^0^	0.00 × 10^0^
F11	Avg	8.94 × 10^−1^	1.92 × 10^−3^	4.75 × 10^−1^	4.82 × 10^0^	9.59 × 10^−1^	8.84 × 10^−1^	0.00 × 10^0^	0.00 × 10^0^
F11	Std	1.10 × 10^−1^	6.08 × 10^−3^	2.48 × 10^−2^	1.09 × 10^0^	5.01 × 10^−2^	7.26 × 10^−2^	0.00 × 10^0^	0.00 × 10^0^
F12	Best	4.93 × 10^2^	1.82 × 10^−19^	8.20 × 10^−1^	1.65 × 10^4^	7.07 × 10^2^	5.71 × 10^2^	6.75 × 10^−91^	4.18 × 10^−123^
F12	Avg	6.30 × 10^2^	1.41 × 10^1^	1.85 × 10^2^	3.23 × 10^4^	8.53 × 10^2^	8.81 × 10^2^	1.70 × 10^−89^	1.80 × 10^−119^
F12	Std	1.25 × 10^2^	1.26 × 10^1^	1.57 × 10^2^	7.22 × 10^3^	1.09 × 10^2^	1.84 × 10^2^	1.70 × 10^−89^	5.28 × 10^−119^
F13	Best	1.31 × 10^4^	3.58 × 10^−1^	1.89 × 10^−1^	2.42 × 10^4^	9.36 × 10^3^	3.42 × 10^3^	4.44 × 10^−16^	4.44 × 10^−16^
F13	Avg	2.10 × 10^4^	2.65 × 10^0^	2.17 × 10^−1^	3.95 × 10^4^	1.24 × 10^4^	5.68 × 10^3^	4.44 × 10^−16^	4.44 × 10^−16^
F13	Std	5.38 × 10^3^	2.80 × 10^0^	1.69 × 10^−2^	7.90 × 10^3^	2.72 × 10^3^	1.53 × 10^3^	3.29 × 10^−32^	0.00 × 10^0^
F14	Best	4.11 × 10^5^	4.64 × 10^1^	4.89 × 10^1^	5.91 × 10^8^	8.43 × 10^4^	4.45 × 10^4^	4.66 × 10^1^	4.51 × 10^1^
F14	Avg	7.98 × 10^5^	4.76 × 10^1^	4.89 × 10^1^	1.98 × 10^9^	3.58 × 10^5^	2.45 × 10^5^	4.71 × 10^1^	4.60 × 10^1^
F14	Std	3.13 × 10^5^	6.26 × 10^−1^	2.01 × 10^−2^	7.01 × 10^8^	2.29 × 10^5^	2.05 × 10^5^	5.54 × 10^−1^	9.35 × 10^−1^
F15	Best	4.87 × 10^2^	1.52 × 10^−1^	4.35 × 10^69^	2.23 × 10^31^	1.09 × 10^13^	1.22 × 10^2^	2.84 × 10^−46^	4.29 × 10^−62^
F15	Avg	1.02 × 10^6^	1.85 × 10^0^	6.62 × 10^72^	1.60 × 10^48^	1.03 × 10^41^	2.46 × 10^2^	1.80 × 10^−45^	5.81 × 10^−61^
F15	Std	3.22 × 10^6^	1.29 × 10^0^	1.07 × 10^73^	5.06 × 10^48^	3.27 × 10^41^	9.17 × 10^1^	1.66 × 10^−45^	1.11 × 10^−60^
F16	Best	4.25 × 10^2^	1.23 × 10^1^	3.30 × 10^2^	1.20 × 10^4^	7.04 × 10^2^	6.02 × 10^2^	9.83 × 10^−39^	1.23 × 10^−53^
F16	Avg	5.60 × 10^2^	2.40 × 10^1^	3.63 × 10^2^	1.47 × 10^4^	8.04 × 10^2^	9.29 × 10^2^	3.72 × 10^−38^	9.51 × 10^−53^
F16	Std	9.66 × 10^1^	1.60 × 10^1^	2.81 × 10^1^	2.84 × 10^3^	9.60 × 10^1^	3.26 × 10^2^	2.18 × 10^−38^	1.37 × 10^−52^
F17	Best	3.79 × 10^1^	3.77 × 10^−12^	2.19 × 10^−1^	9.11 × 10^3^	7.23 × 10^1^	6.41 × 10^1^	4.44 × 10^−16^	4.44 × 10^−16^
F17	Avg	6.75 × 10^1^	7.39 × 10^−12^	2.28 × 10^−1^	1.29 × 10^4^	1.02 × 10^2^	8.04 × 10^1^	4.44 × 10^−16^	4.44 × 10^−16^
F17	Std	3.63 × 10^1^	2.43 × 10^−12^	7.27 × 10^−3^	4.14 × 10^3^	3.19 × 10^1^	1.35 × 10^1^	7.35 × 10^−32^	0.00 × 10^0^
F18	Best	1.38 × 10^4^	1.12 × 10^−2^	9.06 × 10^−2^	2.95 × 10^4^	9.05 × 10^3^	4.19 × 10^3^	7.33 × 10^−24^	2.39 × 10^−46^
F18	Avg	2.29 × 10^4^	2.93 × 10^0^	1.15 × 10^−1^	3.53 × 10^4^	1.32 × 10^4^	6.03 × 10^3^	2.80 × 10^−18^	2.56 × 10^−41^
F18	Std	1.05 × 10^4^	4.53 × 10^0^	1.39 × 10^−2^	4.60 × 10^3^	3.29 × 10^3^	1.60 × 10^3^	8.39 × 10^−18^	7.93 × 10^−41^
F19	Best	1.34 × 10^5^	4.46 × 10^1^	4.89 × 10^1^	1.07 × 10^9^	9.01 × 10^4^	7.63 × 10^4^	4.20 × 10^1^	4.13 × 10^1^
F19	Avg	5.93 × 10^5^	4.70 × 10^1^	4.89 × 10^1^	2.00 × 10^9^	3.74 × 10^5^	2.45 × 10^5^	4.49 × 10^1^	4.28 × 10^1^
F19	Std	4.74 × 10^5^	1.35 × 10^0^	2.82 × 10^−2^	7.49 × 10^8^	3.31 × 10^5^	1.11 × 10^5^	1.82 × 10^0^	9.05 × 10^−1^
F20	Best	3.15 × 10^4^	6.85 × 10^−9^	1.78 × 10^69^	5.19 × 10^31^	2.20 × 10^21^	2.94 × 10^3^	1.77 × 10^−46^	3.18 × 10^−62^
F20	Avg	4.82 × 10^4^	1.57 × 10^1^	7.13 × 10^71^	3.67 × 10^42^	2.19 × 10^39^	6.72 × 10^3^	4.52 × 10^−45^	7.16 × 10^−61^
F20	Std	1.52 × 10^4^	1.03 × 10^1^	1.24 × 10^72^	1.14 × 10^43^	4.62 × 10^39^	2.91 × 10^3^	5.57 × 10^−45^	8.67 × 10^−61^
F21	Best	1.80 × 10^5^	4.89 × 10^1^	4.90 × 10^1^	6.09 × 10^8^	2.10 × 10^5^	1.61 × 10^5^	4.88 × 10^1^	4.88 × 10^1^
F21	Avg	7.05 × 10^5^	4.89 × 10^1^	4.90 × 10^1^	1.91 × 10^9^	4.80 × 10^5^	2.94 × 10^5^	4.88 × 10^1^	4.88 × 10^1^
F21	Std	4.92 × 10^5^	1.20 × 10^−2^	1.12 × 10^−2^	8.08 × 10^8^	3.01 × 10^5^	1.02 × 10^5^	4.42 × 10^−3^	5.73 × 10^−3^
F22	Best	2.38 × 10^4^	2.11 × 10^−9^	1.27 × 10^67^	9.64 × 10^33^	8.52 × 10^26^	9.60 × 10^3^	4.44 × 10^−16^	4.44 × 10^−16^
F22	Avg	2.35 × 10^9^	3.40 × 10^−7^	1.02 × 10^73^	3.04 × 10^45^	3.22 × 10^46^	2.11 × 10^4^	4.44 × 10^−16^	4.44 × 10^−16^
F22	Std	7.42 × 10^9^	6.40 × 10^−7^	2.03 × 10^73^	9.55 × 10^45^	1.02 × 10^47^	2.11 × 10^4^	4.65 × 10^−32^	0.00 × 10^0^
F23	Best	3.64 × 10^1^	5.56 × 10^−12^	2.21 × 10^−1^	6.39 × 10^3^	7.66 × 10^1^	5.49 × 10^1^	4.44 × 10^−16^	4.44 × 10^−16^
F23	Avg	7.64 × 10^1^	9.57 × 10^−12^	2.31 × 10^−1^	1.34 × 10^4^	1.12 × 10^2^	9.46 × 10^1^	4.44 × 10^−16^	4.44 × 10^−16^
F23	Std	4.32 × 10^1^	2.92 × 10^−12^	7.01 × 10^−3^	3.59 × 10^3^	2.81 × 10^1^	1.77 × 10^1^	8.05 × 10^−32^	0.00 × 10^0^
F24	Best	2.40 × 10^5^	4.90 × 10^1^	4.90 × 10^1^	1.14 × 10^9^	9.75 × 10^4^	9.62 × 10^4^	4.89 × 10^1^	4.89 × 10^1^
F24	Avg	7.31 × 10^5^	4.90 × 10^1^	4.90 × 10^1^	2.36 × 10^9^	3.37 × 10^5^	2.37 × 10^5^	4.89 × 10^1^	4.89 × 10^1^
F24	Std	6.72 × 10^5^	2.37 × 10^−3^	4.15 × 10^−3^	1.11 × 10^9^	2.17 × 10^5^	1.40 × 10^5^	4.15 × 10^−4^	5.71 × 10^−4^
F25	Best	7.66 × 10^3^	4.53 × 10^0^	4.52 × 10^70^	1.60 × 10^35^	6.40 × 10^17^	2.85 × 10^3^	2.56 × 10^−46^	2.49 × 10^−62^
F25	Avg	4.02 × 10^4^	2.26 × 10^1^	9.99 × 10^72^	7.65 × 10^46^	1.22 × 10^46^	7.72 × 10^3^	4.82 × 10^−45^	6.15 × 10^−61^
F25	Std	2.24 × 10^4^	1.33 × 10^1^	8.36 × 10^72^	2.42 × 10^47^	3.84 × 10^46^	3.30 × 10^3^	6.05 × 10^−45^	6.97 × 10^−61^
F26	Best	2.31 × 10^4^	1.47 × 10^−12^	5.59 × 10^66^	9.58 × 10^30^	3.51 × 10^14^	2.97 × 10^3^	4.44 × 10^−16^	4.44 × 10^−16^
F26	Avg	4.54 × 10^4^	4.13 × 10^−12^	1.75 × 10^74^	1.21 × 10^42^	1.03 × 10^37^	5.30 × 10^3^	4.44 × 10^−16^	4.44 × 10^−16^
F26	Std	1.58 × 10^4^	2.85 × 10^−12^	5.38 × 10^74^	2.60 × 10^42^	3.08 × 10^37^	2.40 × 10^3^	8.05 × 10^−32^	0.00 × 10^0^
F27	Best	1.39 × 10^4^	−8.91 × 10^−2^	7.88 × 10^−2^	2.41 × 10^4^	9.06 × 10^3^	3.57 × 10^3^	−2.11 × 10^−1^	−2.74 × 10^−1^
F27	Avg	2.70 × 10^4^	3.27 × 10^0^	1.04 × 10^−1^	3.57 × 10^4^	1.26 × 10^4^	6.74 × 10^3^	−1.56 × 10^−1^	−2.09 × 10^−1^
F27	Std	9.61 × 10^3^	3.35 × 10^0^	1.65 × 10^−2^	1.03 × 10^4^	3.49 × 10^3^	2.30 × 10^3^	5.87 × 10^−2^	5.07 × 10^−2^
F28	Best	7.08 × 10^4^	4.81 × 10^1^	4.89 × 10^1^	7.02 × 10^8^	7.39 × 10^4^	1.46 × 10^5^	4.80 × 10^1^	4.79 × 10^1^
F28	Avg	6.61 × 10^5^	4.84 × 10^1^	4.90 × 10^1^	1.64 × 10^9^	4.04 × 10^5^	2.69 × 10^5^	4.82 × 10^1^	4.80 × 10^1^
F28	Std	6.16 × 10^5^	2.02 × 10^−1^	2.28 × 10^−2^	7.06 × 10^8^	3.81 × 10^5^	1.15 × 10^5^	1.25 × 10^−1^	3.19 × 10^−2^
F29	Best	1.13 × 10^5^	9.32 × 10^0^	4.84 × 10^69^	2.57 × 10^33^	1.14 × 10^23^	1.50 × 10^4^	2.68 × 10^−45^	6.45 × 10^−62^
F29	Avg	1.09 × 10^13^	3.38 × 10^1^	1.58 × 10^72^	2.60 × 10^43^	1.39 × 10^42^	5.91 × 10^4^	1.18 × 10^−44^	1.69 × 10^−60^
F29	Std	3.46 × 10^13^	1.45 × 10^1^	3.31 × 10^72^	8.00 × 10^43^	4.40 × 10^42^	6.26 × 10^4^	9.79 × 10^−45^	1.98 × 10^−60^
F30	Best	9.61 × 10^4^	4.83 × 10^1^	4.89 × 10^1^	6.18 × 10^8^	1.10 × 10^5^	8.57 × 10^4^	4.82 × 10^1^	4.82 × 10^1^
F30	Avg	8.95 × 10^5^	4.85 × 10^1^	4.90 × 10^1^	2.19 × 10^9^	3.84 × 10^5^	2.33 × 10^5^	4.83 × 10^1^	4.82 × 10^1^
F30	Std	4.53 × 10^5^	1.56 × 10^−1^	3.31 × 10^−2^	1.05 × 10^9^	3.77 × 10^5^	1.21 × 10^5^	7.55 × 10^−2^	4.77 × 10^−2^

**Table 4:** Differential performance and average rank of CEC2017.

Algorithm	INGO	NGO	SWO	DBO	GTO	BOA	GWO	PSO
Differential expression (Y/N)	0/0	7/1	10/2	12/2	10/3	8/1	10/0	10/1
Aaverage rank	1.25	2.31	5.92	6.6	5.79	5.6	3.04	3.87

**Table 5:** Comparison of 2D coverage effects of eight algorithms.

	PSO	GWO	BOA	GTO	DBO	SWO	NGO	INGO
Best (%)	94.32	96.32	77.16	89.4	94.88	94.2	90.6	98.32
Mean (%)	93.35	95.04	75.976	86.464	93.128	93.176	88.32	97.64
Std (%)	0.92	0.94	0.76	1.87	1.80	1.27	1.54	0.45

**Table 6:** Comparison of 3D coverage effects of eight algorithms.

	PSO	GWO	BOA	GTO	DBO	SWO	NGO	INGO
Best (%)	72.26	74.66	58.59	66.85	74.66	74.31	65.54	72.32
Mean (%)	71.42	73.38	57.32	64.50	73.27	72.81	64.90	70.97
Std (%)	0.70	0.97	0.95	1.39	1.24	1.12	0.51	1.66

## Data Availability

The data that support the findings of this study are available from the corresponding author upon request. There are no restrictions on data availability.
